# How to Steer and Control ERK and the ERK Signaling Cascade Exemplified by Looking at Cardiac Insufficiency

**DOI:** 10.3390/ijms20092179

**Published:** 2019-05-02

**Authors:** Tim Breitenbach, Kristina Lorenz, Thomas Dandekar

**Affiliations:** 1 Biozentrum, Universität Würzburg, Am Hubland, 97074 Würzburg, Germany; dandekar@biozentrum.uni-wuerzburg.de; 2 Institute of Pharmacology and Toxicology, Versbacher Straße 9, 97078 Würzburg, Germany; lorenz@toxi.uni-wuerzburg.de; 3 Leibniz-Institut für Analytische Wissenschaften-ISAS-e.V., 44139 Dortmund, Germany

**Keywords:** optimal pharmacological modulation, efficient intervention points, ERK signaling, optimal treatment strategies, optimal drug targeting, optimal drug combination

## Abstract

Mathematical optimization framework allows the identification of certain nodes within a signaling network. In this work, we analyzed the complex extracellular-signal-regulated kinase 1 and 2 (ERK1/2) cascade in cardiomyocytes using the framework to find efficient adjustment screws for this cascade that is important for cardiomyocyte survival and maladaptive heart muscle growth. We modeled optimal pharmacological intervention points that are beneficial for the heart, but avoid the occurrence of a maladaptive ERK1/2 modification, the autophosphorylation of ERK at threonine 188 (ERK${}^{\mathrm{Thr}188}$ phosphorylation), which causes cardiac hypertrophy. For this purpose, a network of a cardiomyocyte that was fitted to experimental data was equipped with external stimuli that model the pharmacological intervention points. Specifically, two situations were considered. In the first one, the cardiomyocyte was driven to a desired expression level with different treatment strategies. These strategies were quantified with respect to beneficial effects and maleficent side effects and then which one is the best treatment strategy was evaluated. In the second situation, it was shown how to model constitutively activated pathways and how to identify drug targets to obtain a desired activity level that is associated with a healthy state and in contrast to the maleficent expression pattern caused by the constitutively activated pathway. An implementation of the algorithms used for the calculations is also presented in this paper, which simplifies the application of the presented framework for drug targeting, optimal drug combinations and the systematic and automatic search for pharmacological intervention points. The codes were designed such that they can be combined with any mathematical model given by ordinary differential equations.

## 1. Introduction

The Ras/Raf/MEK/ERK1/2 cascade is involved in a panoplyof physiological and pathophysiological processes in the body and is crucial for life. Processes include cell proliferation, cell differentiation, cell hypertrophy and resistance to apoptosis. Even though several mechanisms are known that control the activation of this cascade, a controlled activation of certain functions or selective interference with certain maladaptive functions of the cascade would be of help to use this cascade as pharmacological target.

The Ras/Raf/Mek ERK1/2 cascade has ERK as last amplifier. The Ras/Raf/MEK/ERK1/2 cascade integrates extracellular signals from surface receptors to multiple cellular processes such as gene expression or cell survival. Upstream receptors include receptor tyrosine kinases and G protein coupled receptors. The activation of Raf is induced by the small GTPase Ras; Raf in turn phosphorylates and activates MEK, which then phosphorylate the effector kinases ERK1/2 at the so-called TEY motif leading to the activation of ERK1/2. ERK1/2 phosphorylate several hundred targets [1,2,3] throughout the cytosol and the nucleus. The nuclear/cytosolic distribution of ERK1/2 is controlled by scaffold proteins such as KSR, PEA-15 and Sef and by active and passive nuclear translocation processes involving the nuclear pore [1,2,3].

The Ras/Raf/MEK/ERK1/2 cascade integrates extracellular signals from surface receptors to multiple cellular processes such as gene expression or cell survival. The Ras/Raf/MEK ERK cascade has ERK as last amplifier. Upstream receptors include receptor tyrosine kinases and G protein coupled receptors. The activation of Raf is induced by the small GTPase Ras; Raf in turn phosphorylates and activates MEK, which then phosrphorylates the effector kinases ERK1/2 at the so-called TEY motif leading to the activation of ERK1/2. ERK1/2 phosphorylate multiple targets (>200) throughout the cytosol and the nucleus. There are even more putative ERK substrates known (about 600); however, not all are verified to be functionally relevant [1,2,3]. The nuclear/cytosolic distribution of ERK1/2 is controlled by scaffold proteins such as KSR, PEA-15 and Sef and by active and passive nuclear translocation processes involving the nuclear pore. Further, an autophosphorylation of ERK1/2 has been described that enhances the activation of nuclear ERK1/2 targets. This autophosphorylation at threonine 188 (in ERK2; respectively, threonine 208 in ERK1) can be induced by the activation of GPCRs coupled to G${}_{q}$ or G${}_{s}$ proteins that lead to the activation of the Ras/Raf/MEK/ERK1/2 cascade, dimerization of ERK and the binding of the G${}_{\beta \gamma }$ subunits of G proteins to the ERK dimer. These molecular pre-requisites induce ERK${}^{\mathrm{Thr}188}$ phosphorylation, which in turn enhances nuclear ERK1/2 signaling [1,4,5,6]. The mechanisms, however, of how ERK${}^{\mathrm{Thr}188}$ phosphorylation induces nuclear ERK signaling are still unclear and may involve importins, which have been described to support the import of ERK1/2 into the nucleus [7,8].

This article focusses on the modulation of the molecular events that involve ERK${}^{\mathrm{Thr}188}$ phosphorylation in order to evaluate the integration of the different signaling parameters and to optimize the ERK${}^{\mathrm{Thr}188}$ phosphorylation as therapeutic target [1,2,3,4,5].

Specifically, our calculations refer to data on ERK${}^{\mathrm{Thr}188}$ phosphorylation in cells and transgenic mice that have been described for the ERK2 mutants that are either phosphorylation deficient at threonine188 (ERK2${}^{T188A}$) or simulate the phosphorylation (ERK2${}^{T188D}$); of note, ERK2${}^{T188D}$ purified from *E. coli* is kinase-inactive, which is in contrast to in vivo and cell data.

Importantly, mutations within the ERK cascade are critical for cancer: ERK, the final amplifier, changes any constant signal from above into a constant proliferative signal for the cell [9]. A well-known example is the B-Raf cancer mutation in skin epithelia. This triggers then constant proliferation in melanoma cells, leading to melanoma and treatable well by by a combination of B-Raf and MEK inhibitors (e.g., vemurafenib and cobimetanib). However, resistance is an issue of the treatment with these inhibitors and additional therapeutic options are necessary such as ERK1/2 inhibitors or alternative targeting strategies of protein kinases [10,11].

Control of a kinase cascade is thus of pharmacological interest. In the cells, the Ras/Raf/MEK/ ERK1/2 signaling cascade is controlled by dephosphorylation and inactivation mediated by dual specificity phosphatases, protein-tyrosine specific phosphatases, and protein-serine/threonine phosphatases and scaffold proteins. The interplay of phosphatases and kinases is critical for this cascade [11,12], as phosphatases are important counter players of kinases. However, for practical applications, a major drawback is the limited specificity of the phosphatases, and, for our mathematical model, we decided to use a simplified model system focusing on kinases and their activation or deactivation in the pathway. Hence, phosphatases were only modeled implicitly by the deactivation of the kinase. However, additional signaling components can be implemented in future.

Here, we show a mathematical framework to study ERK signaling and its kinase cascade pinpointed by the examples discussed in the following. Notably, this framework allows calculating how to steer a biological signaling network pharmacologically. We exemplify the approach on ERK and connected cascades as ERK inhibition is of high medical interest. In particular, the third ERK phosphorylation, the ERK${}^{\mathrm{Thr}188}$ phosphorylation, is a precondition for cardiac hypertrophy [13] and thus it is interesting to stimulate signaling pathways in cardiomyocytes that contribute to a proper cardiac function, increase cardiac inotrophy and reduce cardiac remodeling by modulation of this rather maladaptive ERK modification. Finally, as an important applied, point we studied how good the inhibition of the pathological cascade has to be to at least prevent pathological consequences. However, the framework can also be generalized for other signaling cascades, as explained in detail in Supplementary Materials. Moreover, it describes and models all nodes of the whole network considered and this for all time points and all desired pharmacological interventions. Nevertheless, for each real situation, comparison and validation by experimental data is critical and also here the ERK cascade is a nice and well-studied example (e.g., [7,8,9,10,11,14]).

In Section 2.1, we discuss how in principle the provided framework can be used to evaluate different treatment strategies with respect to their beneficial and side effects, important for optimal treatment of chronic disease, such as heart failure. Here, ERK1/2 is shown to be an important target. In Section 2.2, we show how this framework can be used to model constitutively activated pathways, for instance in tumors of the skin such as melanoma. Again, the Ras/Raf/MEK/ERK cascade is critical for cancerogenesis, for instance the mutation of B-Raf (most well known is the ${\mathrm{BRAF}}^{V600E}$ mutation), and then the whole cascade is continuously switched on, with ERK being the final amplifier and the most abundant molecule of the whole cascade. We show with our example of the cardiomyocyte how to use the proposed framework in such a situation to find an optimal treatment strategy out of many possible ones that steers the pathological expression pattern, caused by the constitutively activated pathway, to a desired physiological one. Our basal example in this work is a model of a cardiomyocyte that was fitted to experimental data from [14]. By the fitting to experimental data, the network was analyzed with respect to thresholds in the ERK signaling pathway.

**Figure 1:**
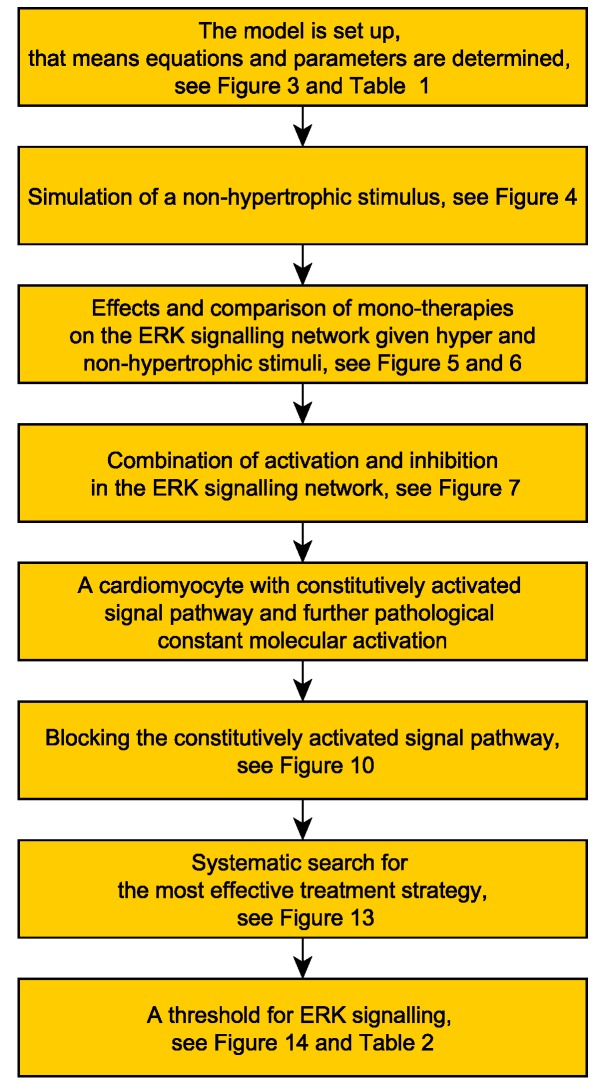
Flow chart of the results.

For a summary of the content of the present paper, see the analysis flow chart in Figure 1 and, for an overview of the methods of the presented framework for finding optimal drug combinations and effective pharmacological intervention points, see Section 4.

## 2. Results

In the following flow chart, we summarize the analysis flow and how the results build on each other.

### 2.1. ERK is of Pivotal Significance in the Cardiomyocyte’s Regulatory Network

ERK1/2 have shown to be essential but at the same time detrimental to the heart: ERK1/2 mediate cell survival but can also mediate cardiomyocyte hypertrophy associated with maladaptive remodeling of the heart and impaired cardiac function. The selective prevention of ERK1/2-mediated cardiac hypertrophy—but not of ERK1/2-mediated cell survival—is thus of interest for the prevention of heart failure [6].

**Figure 2:**
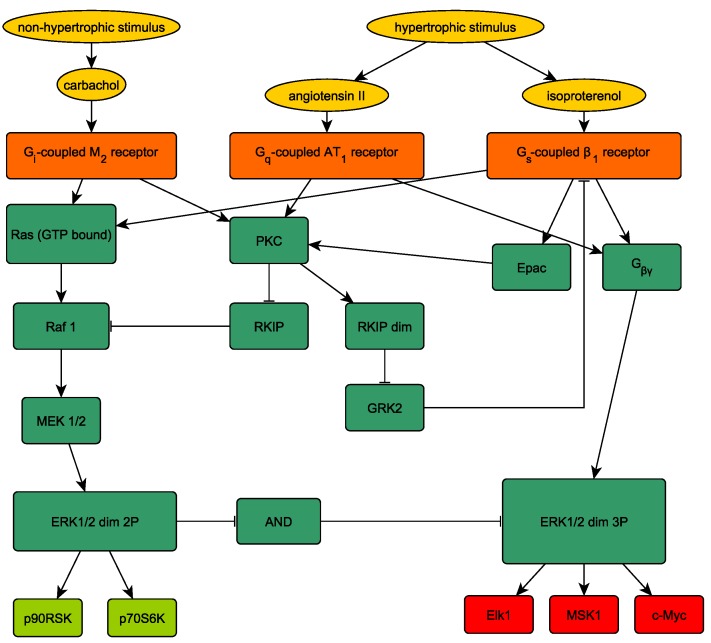
The graph of a network associated with a cardiomyocyte. The Network topology is shown (as in [14] (Figure 1), which includes also references and explanations for all involved nodes and their interactions; colors are different): In orange, the non-hypertrophic stimulus (left, carbachol) or the hypertrophic stimulus (either angiotensin II or isoproterenol) are active after a suitable stimulus (e.g., stress, oxygen debt of the heart, hormonal input). In green, the network of activations (arrows) and inhibitions (blunted arrows) is shown. The output is positive strengthening of the heart (left, light green), or the pathological hypertrophy of the heart (right, red boxes), the increase in the cardiac hypertrophy is mediated by the activation of transcription factors entering the nuclei of the cardiomyocytes, three important ones are shown.

In this section, we consider a gene regulatory network for cardiomyocytes given in [14] where we discuss how to use the presented framework in principle to calculate different strategies to act on this network with external stimuli and to find out optimal pharmacological targets. A strategy is defined as which nodes we have to activate or inhibit by external stimuli. The network’s graph is shown in Figure 2. Here, we would like to make another point: A model is never accurate and hence there are always additions you can consider in a more complex model. For example, Epac does not seem to activate PKC, but rather it can activate Ras. However, we start with a simpler model as we aim to give a general framework for network and drug target analyzing. On the one hand, the framework is for any model that is set up of differential equations and not fixed to the model depicted in Figure 2. For a practical use, we provide the corresponding Matlab scripts where the user just has to change the corresponding model equations that can also be generated with Jimena [15], SQUAD [16] or Potterswheel [17].

Hence, for explaining the ERK cascade, we use the underlying mathematical model (2.1) in the Supplementary Materials if not otherwise stated where h=10 and $\omega_{k}$, $k\in \left\{1,...,26\right\}$, which are taken from [14] and are given as in Table 1. The nodes AND and SYSTEM STATE are technical nodes, which we describe in the following. The node SYSTEM STATE is not in the network but is used to permanently activate RKIP and GRK2 to model their constitutive expression in our model of a cardiomyocyte. Furthermore, the node SYSTEM STATE activates the node AND such that we have that ERK1/2 dim 3P can only be activated if ERK1/2 dim and $G_{\beta \gamma }$ is active at the same time since the three times phosphorylated ERK1/2 requires the twice phosphorylated ERK1/2. This models the fact that the interdependence between ERK1/2 dimer with two phosphorylations from the positive inotropic cascade and the ERK1/2 dimer with three phosphorylations is represented by an “AND” connection. The equations for node 1, node 10 and node 26 are given as $\frac{dx_{1}}{dt}=-x_{1}$, $\frac{dx_{10}}{dt}=-x_{10}$, which can be supplemented by activating stimuli according to model (2.1) in the Supplementary Materials and $\frac{dx_{26}}{dt}=1-x_{26}$ that ensures that the activity level of SYSTEM STATE is constantly one, which means is set on, to activate node 14, node 16 and node 17.

The control strategy is defined for each experiment separately. We have for the parameters $\sigma_{kj}=\zeta_{kj}=0$ if the external stimulus $u_{j}$ has no effect on the node *k*, $\sigma_{kj}=1$ if the external stimulus has an activating effect on the node *k* and $\zeta_{kj}=1$ if external stimulus has an inhibiting effect on node *k*. It is stated in this work if we use different values for the parameters than these ones. Thus, now we should consider pharmacological knowledge to think about optimal therapy strategies regarding the ERK cascade:

In our case, we associate a high activity of the nodes p90RSK (node 8) and p70S6K (node 9) with beneficial effects and a high activity of the nodes Elk1 (node 23), MSK1 (node 24) and c-Myc (node 25) with maleficent effects.The activity levels of nodes, which ranges in our work between zero and one, stand for the biological production activities of the associated biological agents. For instance, transcription or translation rates of the associated node is gene. In this case, zero is associated with no production and is interpolated until one which represents the highest production rate that is biologically possible.

From our considerations, we desire a high activity for p90RSK and p70S6K and a low activity for Elk1, MSK1 and c-Myc. We define these five nodes as our nodes of interest and choose the desired state for the first two ones constant one and for the last three constant zero. The weights $g_{k}$ for the first two are equal to $\frac{3}{2}$ for the other three equal to 1 to compensate the fact that we have two beneficial nodes and three maleficent ones and thus give the beneficial effect altogether the same weight as the maleficent effect. For our experiments, we always had $x_{0}$ the constant zero vector except the last entry was set to 1, which is the initial value of the system state. To calculate the pharmacological intervention in order to obtain the desired activity pattern of the nodes, we used Algorithm 1 in the Supplementary Materials where we set its parameters as follows numMax=10. If we used fewer than 10 possible external stimuli, which are the intervention possibilities in the lab, then we set numMax to at most the number of used external stimuli. Furthermore, we set numInt=3. The result from these calculations can be used as an initial guess for the sequential Hamiltonian (SQH) method [18] (Algorithm 2) to calculate the fine-tuning for the external stimuli. For this purpose, we use the recommended parameter values from [18] except $\kappa =10^{-14}$ and α=0 if not otherwise stated. The final time is chosen by T=20 which means that the simulation time for the regulatory network is 20 time units. We compare pharmacological treatment strategies as follows.

For our first experiment, we wanted to study the effects of carbachol, angiotensin II and isoproterenol. These are drugs typically used in the clinic to treat high blood pressure (angiotensin 1 receptor blockers) and heart failure (beta receptor blockade) and are, in particular in older, multi-morbid patients, often used in combination. Thus, translated into the mathematical language of our framework, this reads as follows.

**Figure 3:**
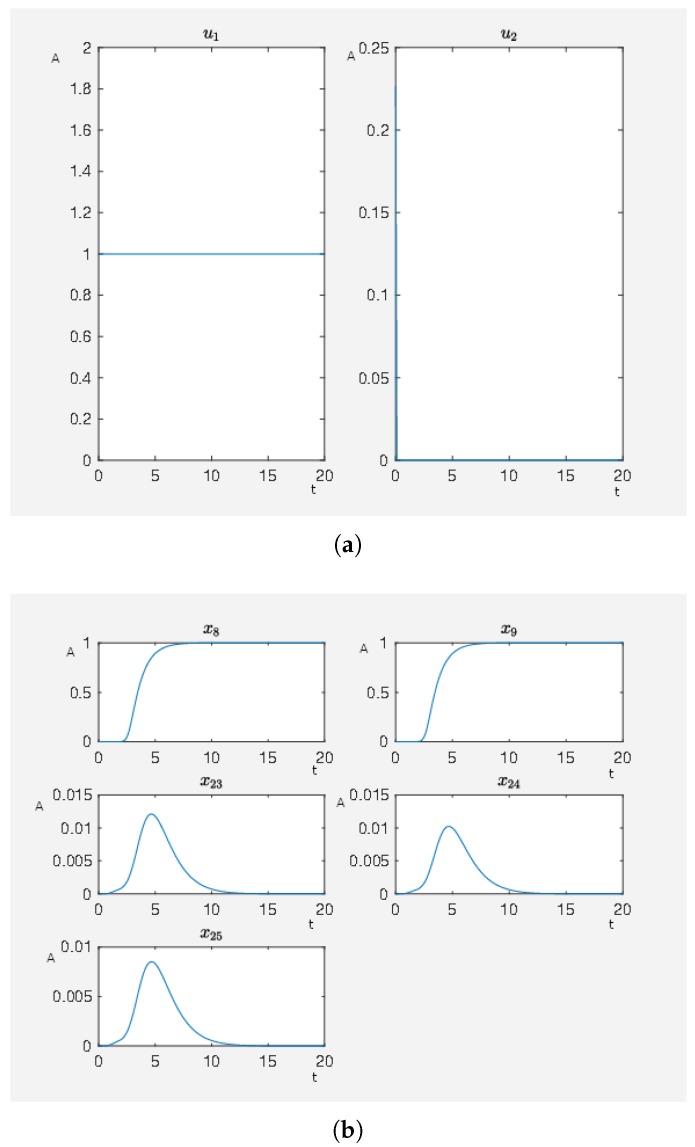
A non-hypertrophic stimulus with carbachol and angiotensin II. (**a**) Time curve of the external stimuli where $u_{1}$ activates carbachol and $u_{2}$ angiotensin II. On the abscissa, we have the time (t) and on the ordinate the activity level (A). (**b**) Time curve of the nodes of interests where $x_{8}$ is the activity level of p90RSK, $x_{9}$ is the activity level of p70S6K, $x_{23}$ is the activity level of Elk1, $x_{24}$ is the activity level of MSK1 and $x_{25}$ is the activity level of c-Myc. On the abscissa, we have the time (t) and on the ordinate the activity level (A).

We have an activating external stimulus on the node carbachol, angiotensin II and isoproterenol. When the SQH method stops and returns a solution, we have $J_{0}=4.802759$ and, in Figure 3, we can see the time curves of the external stimuli, which are not the zero function and the time curves of the states of interest. We see that an activation of carbachol leads to the activation of the beneficial nodes. The short pulse of angiotensin II supports this effect and the maleficent nodes decay after a short and weak activation.

On the other hand, our framework can be used to check if a model is reasonable by testing what happens subject to different external stimuli and if the reactions are in correspondence to experimental data. Remember, the phosphorylation of p70S6K by ERK does not cause the direct activation of the latter and further regulatory events may activate p70S6K. However, everything can be modeled in more detail, considering further or alternative regulatory events. In fact, which important effects to consider depends on the choice of the user. The effects in Figure 3 are a result of the calculation to steer the network depicted in Figure 2 to the desired state. We aimed as follows. By our straightforward framework, we can evaluate models by checking if external stimuli provide known results. In this way, we can easily check if a model is reasonable by testing different situations of external stimuli and their corresponding effects. A more detailed model would refine the model output shown in Figure 3.

#### 2.1.1. Studying the Effects of Mono-Therapy on the ERK Signaling Network

**Figure 4:**
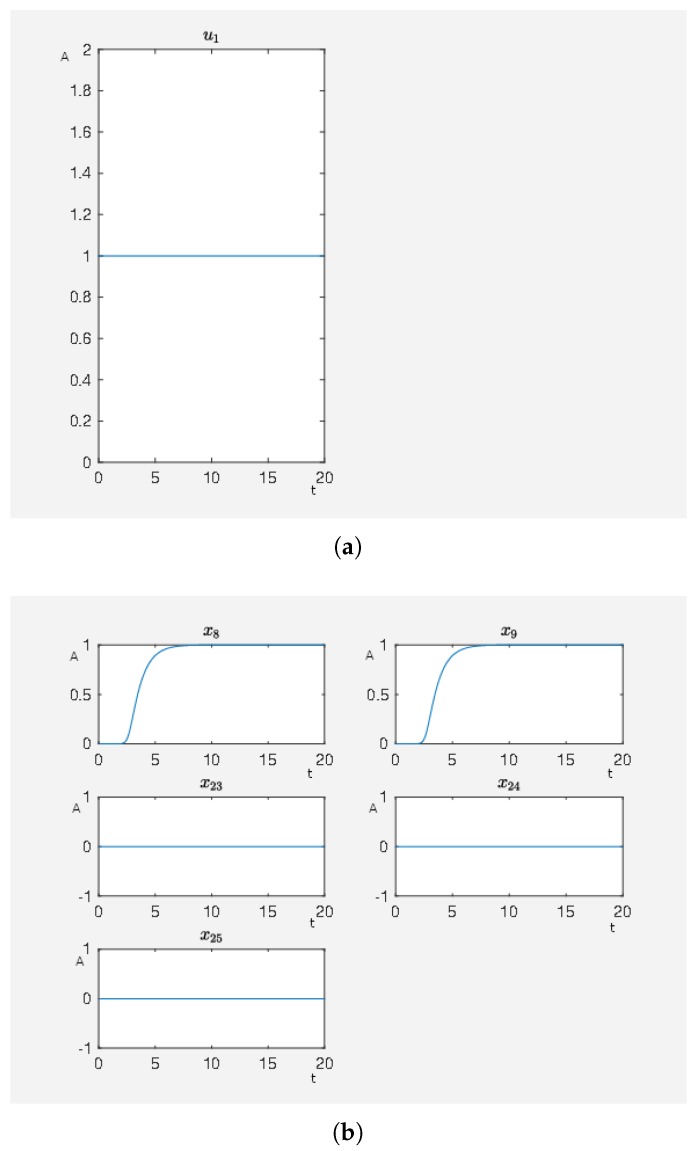
A non-hypertrophic stimulus with carbachol. (**a**) Time curve of the external stimulus where $u_{1}$ activates carbachol. On the abscissa, we have the time (t) and on the ordinate the activity level (A). (**b**) Time curve of the nodes of interests where $x_{8}$ is the activity level of p90RSK, $x_{9}$ is the activity level of p70S6K, $x_{23}$ is the activity level of Elk1, $x_{24}$ is the activity level of MSK1 and $x_{25}$ is the activity level of c-Myc. On the abscissa, we have the time (t) and on the ordinate the activity level (A).

In Figure 4, we see the result where we only have an activating external stimulus on carbachol. The target functional value $J_{0}=4.805384$ when the SQH method converges. If we compare the target functional value with the first experiment, we see that it is just a bit bigger and thus both control strategies can be seen as equivalent control strategies with respect to an activity level close to the desired one.

**Figure 5:**
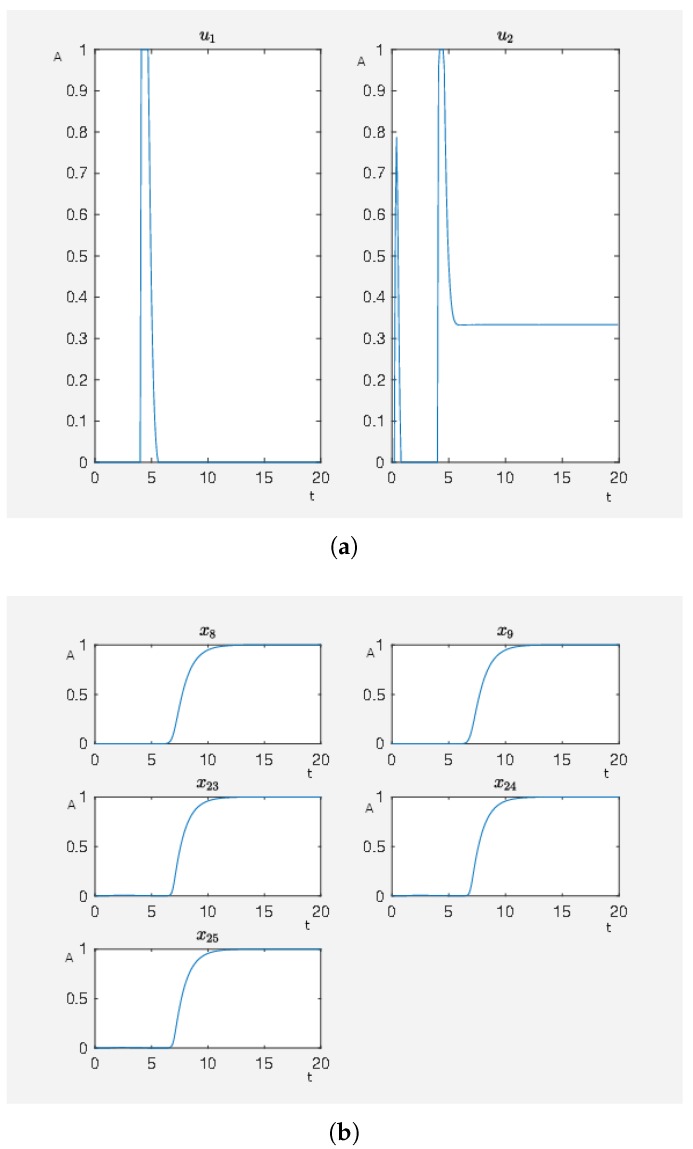
A hypertrophic stimulus with angiotensin II and isoproterenol. (**a**) Time curve of the external stimuli where $u_{1}$ activates angiotensin II and $u_{2}$ isoproterenol. On the abscissa, we have the time (t) and on the ordinate the activity level (A). (**b**) Time curve of the nodes of interests where $x_{8}$ is the activity level of p90RSK, $x_{9}$ is the activity level of p70S6K, $x_{23}$ is the activity level of Elk1, $x_{24}$ is the activity level of MSK1 and $x_{25}$ is the activity level of c-Myc. On the abscissa, we have the time (t) and on the ordinate the activity level (A).

In our third experiment, we had an activating external stimulus just on angiotensin II and isoproterenol. This would correspond to a simultaneous pharmacological treatment with angiotensin II and isoproterenol. When the SQH method converges, we have $J_{0}=28.70478$. In Figure 5, we have the time curves of the external stimuli which are not zero and of the nodes of interest. Compared with the two other experiments, the target functional was much higher, which means that an activating external stimulus on carbachol was essential for an activity level of the network’s nodes of interest close to our desired activity level. By this, we would then avoid heart hypertrophy and generate a strong non-hypertrophic stimulus though.

#### 2.1.2. Combined Effects of Activation and Inhibition on ERK Signaling

ERK${}^{\mathrm{Thr}188}$ autophosphorylation triggers cardiac hypertrophy and subsequent maladaptive remodeling and cardiac insufficiency. Since modulation of ERK${}^{\mathrm{Thr}188}$ phosphorylation does not affect ERK1/2 mediated cell survival, ERK${}^{\mathrm{Thr}188}$ phosphorylation is thought to be an elegant target to intervene with maladaptive ERK1/2 signaling in the heart.

**Figure 6:**
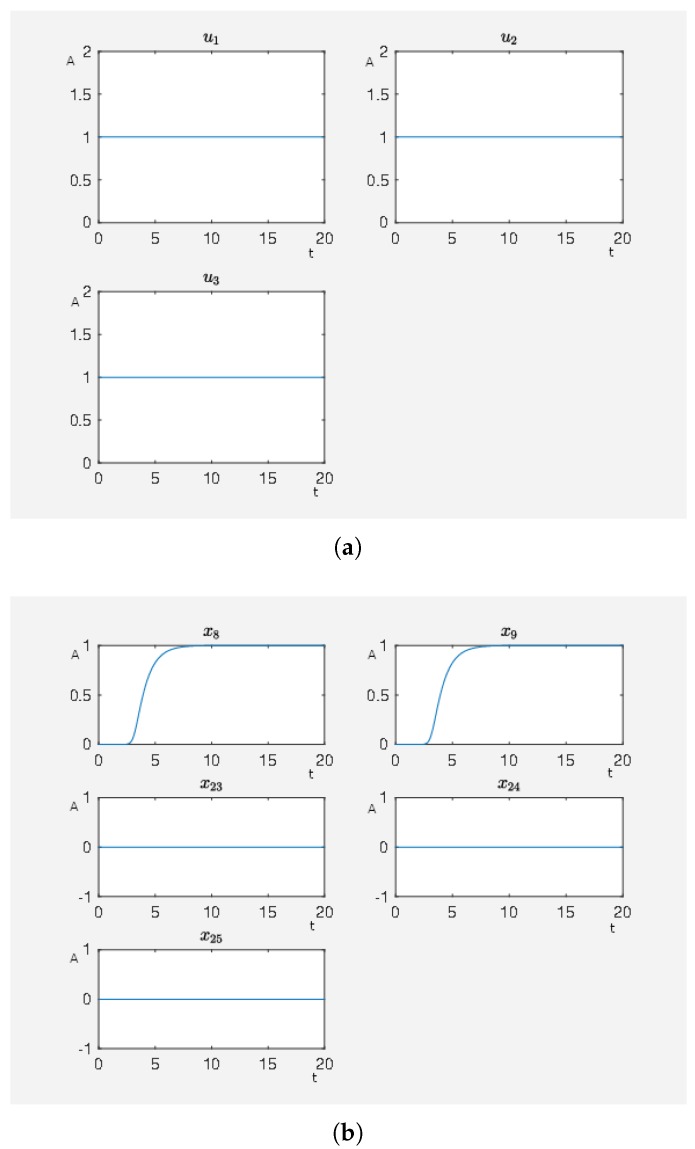
A non-hypertrophic stimulus by angiotensin II and isoproterenol with the inhibition of the third ERK1/2 ERK${}^{\mathrm{Thr}188}$ phosphorylation. (**a**) Time curve of the external stimuli where $u_{1}$ activates angiotensin II, $u_{2}$ on isoproterenol and $u_{3}$ inhibits ERK1/2 dim 3P. On the abscissa, we have the time (t) and on the ordinate the activity level (A). (**b**) Time curve of the nodes of interests where $x_{8}$ is the activity level of p90RSK, $x_{9}$ is the activity level of p70S6K, $x_{23}$ is the activity level of Elk1, $x_{24}$ is the activity level of MSK1 and $x_{25}$ is the activity level of c-Myc. On the abscissa, we have the time (t) and on the ordinate the activity level (A).

In our fourth experiment, we had activating external stimuli on angiotensin II and isoproterenol and one inhibiting external stimulus on ERK1/2 dim 3P. When the SQH method converges, we have $J_{0}=5.513235$ and the corresponding time curves are shown in Figure 6. As the target functional value is close to the one with the experiments where carbachol is activated, we can say that the strategy of activating angiotensin II and isoproterenol while inhibiting ERK1/2 dim 3P is equivalent to the one where we only activate carbachol.

**Figure 7:**
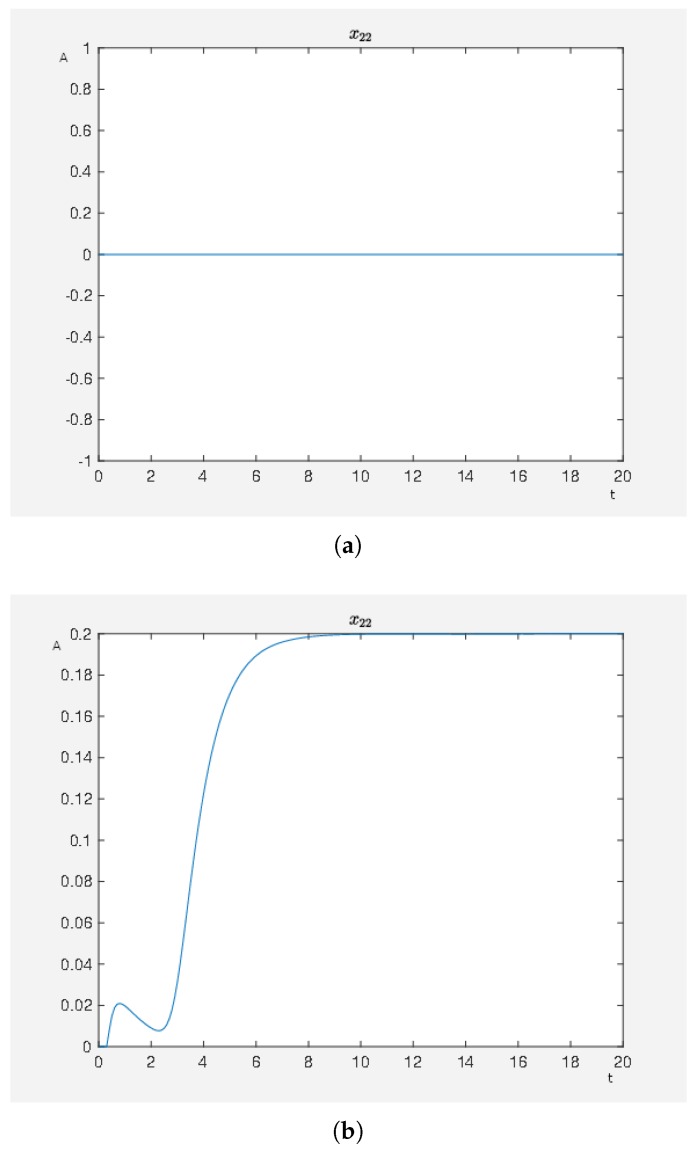
On the abscissa, we have the time (t) and on the ordinate the activity level (A) of the ERK1/2 dim 3P in the forth experiment: (**a**) with $\zeta_{22,3}=1$; and (**b**) with $\zeta_{22,3}=0.8$ in model (2.1) of the Supplementary Materials, which demonstrates how to adapt different capabilities of the external stimuli with respect to influencing the corresponding nodes.

In Figure 7a, we have the activity level of ERK1/2 dim 3P, which is of course an extreme case as we have a very strong inhibition. However, it demonstrates that for a strong inhibition of the ERK1/2 dim 3P that this strategy is almost that good as using an activating stimulus only on carbachol, see the first two experiments.

It is possible to fit an external stimulus’s ability for inhibition by the parameter $\zeta_{kj}$ in (2.1) of the Supplementary Materials such that the corresponding node has the measured activity level when the inhibitor is active. For this purpose, the parameter $\zeta_{22,3}$ can be diminished and thus the activity level of ERK1/2 dim 3P increases for $\zeta_{22,3}$ tending to zero, see Figure 7b.

These first four experiments demonstrated how our optimization framework can be used to compare different control strategies with respect to their ability to steer the activity level to the desired activation level of the network’s nodes. Once the parameters such as *T* and weights $g_{k}$ are fixed (see model (2.2) in the Supplementary Materials), then the smaller the target functional value of a certain control strategy is, the more beneficial effects and the less maleficent effects the strategy has. By this procedure, we can sort different strategy or assess them with respect to their corresponding target functional value. The optimization framework serves as an objective method to determine the time curves of the external stimuli such that we have the lowest target functional value possible for the given strategy. We stress that we just influence on which node an external stimuli acts. Each time curve is then automatically given by the optimization framework, namely by solving model (2.2) in the Supplementary Materials.

In the next two sections, we show how to use our framework to model constitutively activated signal pathways since they play an important role in heart failure and for oncogenesis in general. Then, in the following, we show how to utilize the framework to systematically search for optimal treatment strategies, again with our basal model of a cardiomyocyte.

### 2.2. A Cardiomyocyte with Constitutively Activated $G_{s}$-Coupled $\beta_{1}$ Receptor

**Figure 8:**
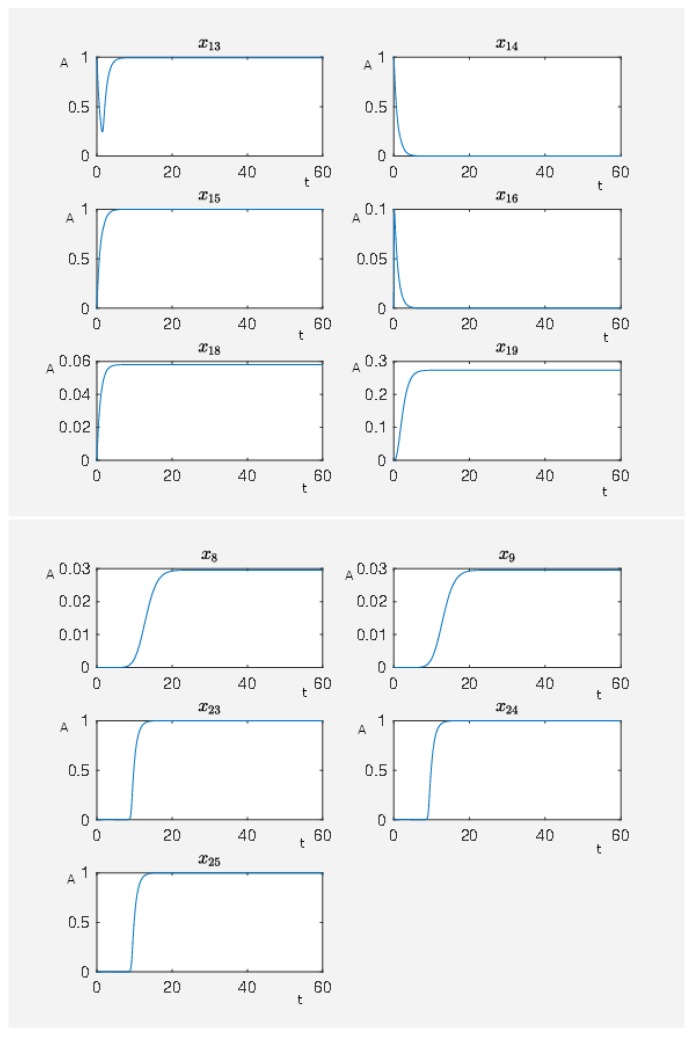
On the abscissa, we have the time (t) and on the ordinate the activity level (A) for PKC (node 13), RKIP (node 14), RKIP dim (node 15), GRK2 (node 16), isoproterenol (node 18), $G_{s}$-coupled $\beta_{1}$ receptor (node 19), p90RSK (node 8), p70S6K (node 9), Elk1 (node 23), MSK1 (node 24), and c-Myc (node 25).

This situation can arise from different situations in real life: For instance, taking constantly a beta-mimetic drug to treat asthma could lead to such a constant activation of the $G_{s}$-coupled $\beta_{1}$ receptor. Alternatively, endogenous factors such as constant stress or first signs of cardiac failure can lead to such an activation. Mathematically speaking, we hence discuss different strategies for the network from Section 2.1 where the $G_{s}$-coupled $\beta_{1}$ receptor (node 19) is constitutively activated such that it has continuously about 30% of its maximum activation level. To model this, we equip its corresponding activating node isoproterenol (node 18) with the term $+0.058-x_{18}$ such that the corresponding equation is given by $\frac{dx_{18}}{dt}=0.058-x_{18}$. Furthermore, in our experiment, node 10 (hypertrophic stimulus) was not activated and thus stayed at zero if an initial value of zero was chosen. This ensured that isoproterenol stayed at a constant level of 5.8% of its maximum activation, which had the consequence that the $G_{s}$-coupled $\beta_{1}$ receptor had about 30% of its maximum activation level (see Figure 8). As in Section 2.1, we associate a high activity of the nodes p90RSK (node 8) and p70S6K (node 9) with beneficial effects and a high activity of the nodes Elk1 (node 23), MSK1 (node 24) and c-Myc (node 25) with maleficent effects, which is why we desire a low activity for them. We define these five nodes as our nodes of interest and choose the desired state for the first two constants one and for the last three constants zero. The weights $g_{k}$ for the the first two are again equal to $\frac{3}{2}$ for the other three equal to 1. We always have $x_{0}$ equal to the constant zero vector except the activity level for AND, GRK2 and SYSTEM STATE equal 1 and we use Algorithm 1 in the Supplementary Materials where we set the algorithm’s parameters as follows numMax=10 and numInt=3. The result from this calculation was used as the initial guess for the sequential Hamiltonian (SQH) method [18] (Algorithm 2) to calculate the fine tuning for the relevant intervention points. The SQH method was used with its recommended parameter values, see [18], except $\kappa =10^{-14}$ and α=0 if not otherwise stated. The final time was chosen by T=60, i.e., the regulatory network was simulated for 60 time units.

If the network is unperturbed, then the constitutively activated $G_{s}$-coupled $\beta_{1}$ receptor causes the following activity pattern in the network, where we show the activity level of some nodes in Figure 8 with $J_{0}=160.1896$. We see that the activity level of the nodes associated with maleficent effects (nodes 23–25) are highly active while the nodes associated with beneficial effects (nodes 8 and 9) are at a very low activity level. Furthermore, we see that a constitutively activated receptor is able to hold the network in a certain state that means a constant expression pattern. Thus, the expression pattern of the network is also constitutively altered compared to the steady state in which the network would be if the receptor was totally inactive.

#### 2.2.1. Further Pathological Constant Molecular Activation

In this subsection, we would like to look at the long-term consequences of the constant activation of the ERK cascade by the constitutively activated $G_{s}$-coupled $\beta_{1}$ receptor. In particular, the continuous activation of a receptor or ERK kinase or other members of the ERK cascade such as MEK may also lead to cancer.

This can also easily be investigated within our framework. In such situations, ERK is part of the signaling cascade while the constitutive activation may either stem from an activating, oncogenic mutation of a key receptor such as Epidermal Growth Factor Receptor (EGFR, usually treated by Gefitinib [19]) or by a kinase mutation (most well known are B-Raf and Ras mutations, however, in some aggressive cancers this can also be ERK mutations).

In general, a constitutively activation of receptors can be caused by mutations in the receptor itself or its corresponding signal protein. Another example is cell–cell-interaction where a constitutively activated receptor can be caused by secretory cells that constitutively secret the corresponding signal molecule. The presented framework in combination with constitutively activated receptors can also be used in modeling oncogenesis where constitutively activated pathways play a role [20,21,22].

A further example is the inhibition of p53 or Retinoblastoma (Rb) protein after a virus infection. This can be caused by constitutively expressed proteins that bind to p53 or Rb to enhance cell proliferation which is needed for the virus reproduction [23,24,25]. This can be modeled analogous to the constitutively activated receptors where the other way round the activity level of the corresponding node is constitutively inhibited by the external stimuli associated with this effect of the virus infection. This illustrates that an external stimulus can also be a virus or the effect of its infection, as well as how this can be modeled within the presented framework. Once the corresponding issue is modeled as described before, the benefit for pharmacological research is the identification of promising drug targets that counter the maleficent effects caused by the constitutively activated pathway. This illustrates how the mechanism modeling constitutively activated $G_{s}$-coupled $\beta_{1}$ receptor as discussed above transfers to different situations and signaling pathways.

#### 2.2.2. Steering the ERK Network of the Cardiomyocyte in a Beneficial Way

Molecular pre-requisites for ERK${}^{\mathrm{Thr}188}$ phosphorylation are the dimerization of ERK and G${}_{\beta \gamma }$ binding to the ERK dimer. Interference with these protein-protein interactions would thus facilitate selective inhibition of ERK${}^{\mathrm{Thr}188}$ phosphorylation. In this manuscript, we particularly evaluate the outcome of the interference with the ERK–ERK interaction in the context of the different ERK1/2 activating cascades.

Now, we show how to apply our framework to discuss different strategies that improve the expression pattern that means to obtain a non-hypertrophic stimulus. First, we show the effects of just blocking the constitutively activated $G_{s}$-coupled $\beta_{1}$ receptor and then we demonstrate how to use our framework to automatically search for an alternative treatment strategy.

**Figure 9:**
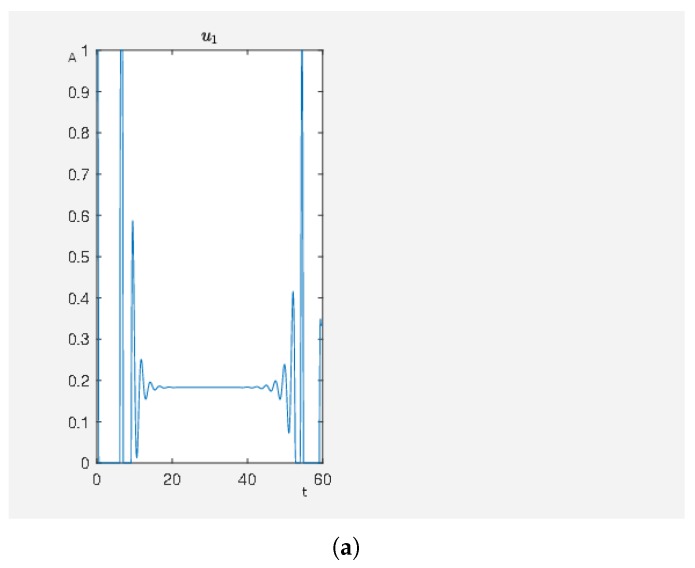

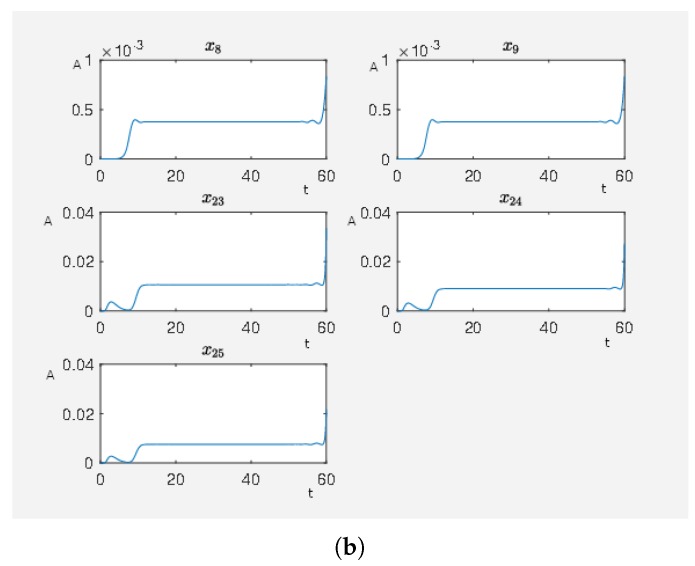
Blocking the constitutively activated G${}_{s}$-coupled $\beta_{1}$ receptor. (**a**) The external stimulus $u_{1}$ inhibits G${}_{s}$-coupled $\beta_{1}$ receptor. On the abscissa, we have the time (t) and on the ordinate the activity level (A). (**b**) Time curve of the nodes of interests where $x_{8}$ is the activity level of p90RSK, $x_{9}$ is the activity level of p70S6K, $x_{23}$ is the activity level of Elk1, $x_{24}$ is the activity level of MSK1 and $x_{25}$ is the activity level of c-Myc. On the abscissa, we have the time (t) and on the ordinate the activity level (A).

**Figure 10:**
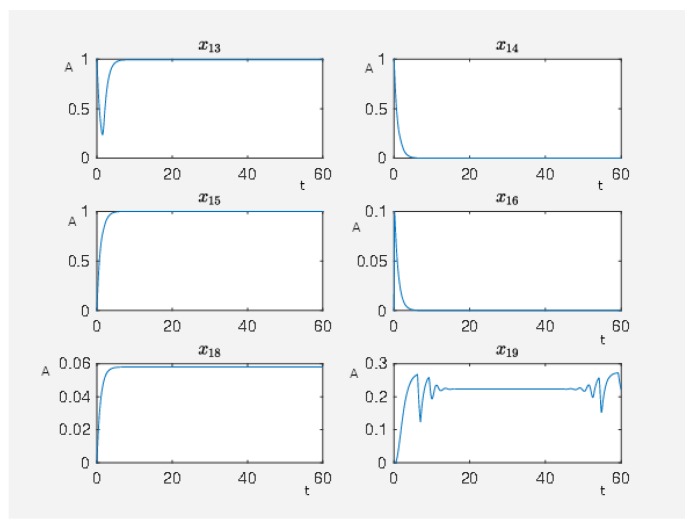
On the abscissa, we have the time (t) and on the ordinate the activity level (A) for PKC (node 13), RKIP (node 14), RKIP dim (node 15), GRK2 (node 16), isoproterenol (node 18) and G${}_{s}$-coupled $\beta_{1}$ receptor (node 19) where the G${}_{s}$-coupled $\beta_{1}$ receptor is inhibited.

A strategy to reduce the target functional value, which means that it increases the beneficial effects, is to inhibit the $G_{s}$-coupled $\beta_{1}$ receptor, which is called the β-block strategy in this work. When the SQH method converges, we have $J_{0}=90.09628$. The results are shown in Figure 9 and Figure 10 for some activity levels of nodes. The time curve of the corresponding external stimuli might be a delicate issue in a real experiment. With a constant external stimulus with value 0.2, we have the value of the cost functional $J_{0}=90.10581$. Therefore, it is not needed to have such a highly structured time curve, as shown in Figure 9, because we obtain the same order of magnitude of the target function with the corresponding constant external stimulus. Notice that it is sufficient to reduce the activity level of the $G_{s}$-coupled $\beta_{1}$ receptor from about 30% to about 24% such that the activity level of Elk1, MSK1 and c-Myc drops from about 100% to 1%. That means that we have identified a threshold for the activity of these three nodes via the activation of the G${}_{s}$-coupled $\beta_{1}$ receptor. In this way, we can use the framework to calculate a fine tuning of nodes. By equipping a node by an external stimulus, we see after the calculation how much the activity level upon the action of the external stimulus differs from the unperturbed situation.

#### 2.2.3. Systematic Search for an Optimal Treatment

For clinical applications, steering the ERK signaling pathway is critical. This is achieved usually by pharmacological drugs. However, ERK is in a network and rarely the complete network response is considered. For this reason, we discuss in the following the network effects, too.

**Figure 11:**
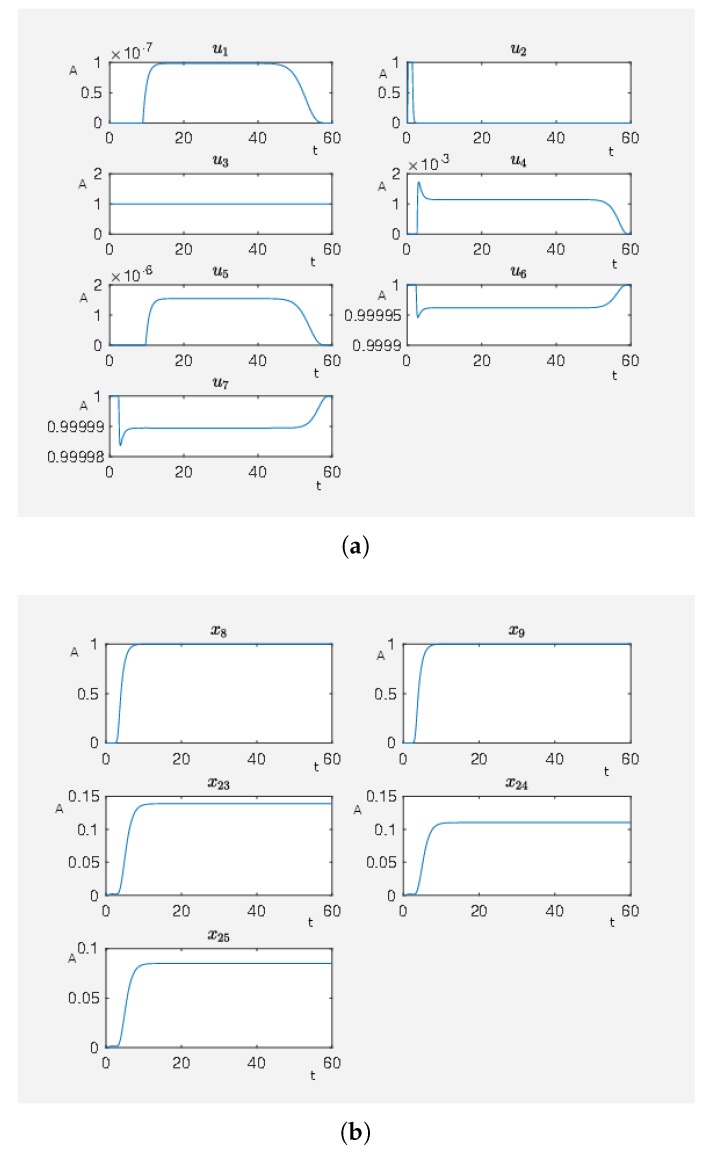
Several external stimuli acting on the cardiomyocyte. (**a**) Time curve of external stimuli where $u_{1}$ inhibits PKC, $u_{2}$ inhibits RKIP and RKIP dim, $u_{3}$ inhibits ERK1/2 dim 3P, $u_{4}$ G${}_{s}$-coupled $\beta_{1}$ receptor, $u_{5}$ inhibits Ras (GTP bound), $u_{6}$ activates angiotensin II and $u_{7}$ activates isoproterenol. On the abscissa, we have the time (t) and on the ordinate the activity level (A). (**b**) Time curve of the nodes of interests where $x_{8}$ is the activity level of p90RSK, $x_{9}$ is the activity level of p70S6K, $x_{23}$ is the activity level of Elk1, $x_{24}$ is the activity level of MSK1 and $x_{25}$ is the activity level of c-Myc. On the abscissa, we have the time (t) and on the ordinate the activity level (A).

To obtain a further improvement of the therapy, i.e., more beneficial effects and fewer side effects, we now perform a systematic search for beneficial intervention points. In mathematical terms, it means that we find external stimuli that effect the activity levels of certain nodes and are associated with a therapy that reduces the target functional, defined in (2.2) in the Supplementary Materials, which takes maleficent side effects and beneficial effects of a therapy via the corresponding external stimuli into account. In the case of a small target functional value, we have external stimuli that steer the considered network to a state with corresponding activity levels of the nodes that are associated with a healthy state. For our systematic search, we equip the nodes PKC ($u_{1}$), RKIP ($u_{2}$), RKIP dim ($u_{2}$), ERK1/2 dim 3P ($u_{3}$), G${}_{s}$-coupled $\beta_{1}$ receptor ($u_{4}$) and Ras (GTP bound) ($u_{5}$) with inhibiting external stimuli and the nodes angiotensin II ($u_{6}$) and isoproterenol ($u_{7}$) with activating external stimuli. In our example, we set $\zeta_{22,3}=0.95$ to model the fact that possibly the external stimuli $u_{3}$ cannot totally inactivate of ERK1/2 dim 3P. In Figure 11, we can see the results. Notice that we now have high activity levels for the nodes p90RSK and p70S6K and still a low activity level for the nodes Elk1, MSK1 and c-Myc which results in a lower target functional value than in the last experiment. We have a target functional value $J_{0}=6.594448$. That means this treatment is better than just blocking the G${}_{s}$-coupled $\beta_{1}$ receptor. However, there are many external stimuli active. Our next step is to use our framework to reduce the number of external stimuli in order to obtain the most effective external stimuli. These are the external ones to focus on for designing an optimal therapy.

**Figure 12:**
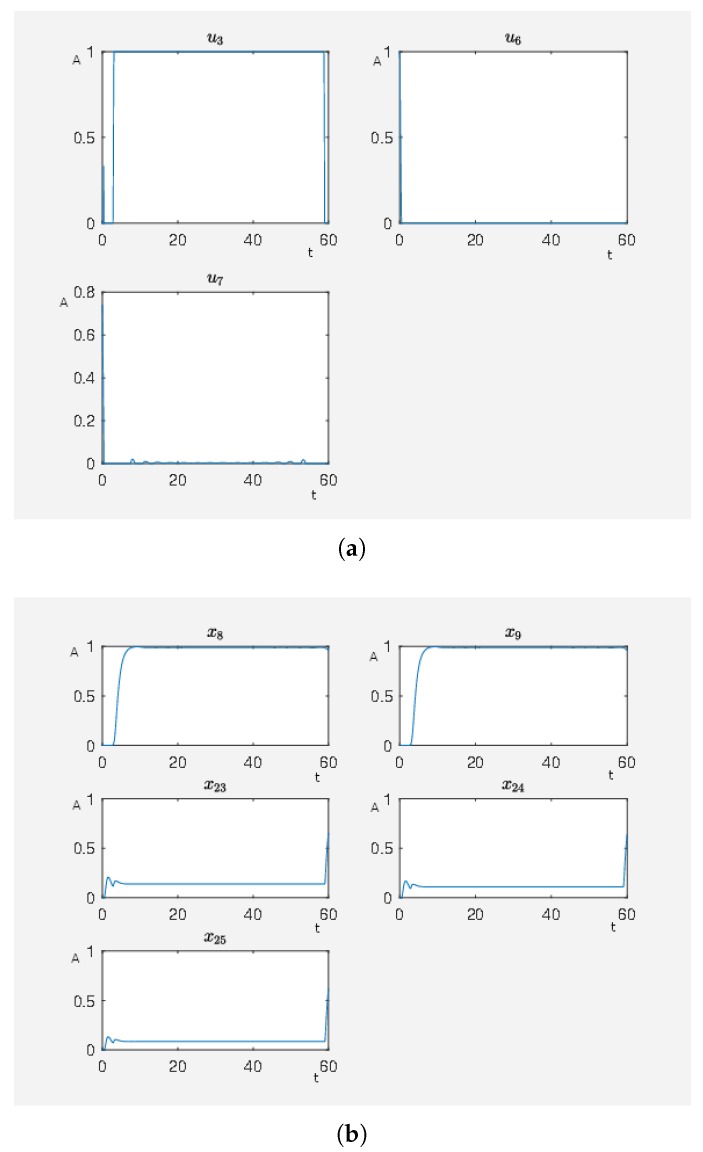
The optimal treatment strategy. (**a**) Time curve of external stimuli where $u_{3}$ inhibits ERK1/2 dim 3P, $u_{6}$ activates angiotensin II and $u_{7}$ activates isoproterenol. On the abscissa, we have the time (t) and on the ordinate the activity level (A). (**b**) Time curve of the nodes of interests where $x_{8}$ is the activity level of p90RSK, $x_{9}$ is the activity level of p70S6K, $x_{23}$ is the activity level of Elk1, $x_{24}$ is the activity level of MSK1 and $x_{25}$ is the activity level of c-Myc. On the abscissa, we have the time (t) and on the ordinate the activity level (A).

In Figure 11, there are many active external stimuli. By increasing α>0 in model (2.2) of the Supplementary Materials, we reduce the number of active external stimuli. This comes from the fact that by increasing alpha the costs for active external stimuli increase such that only that ones remain whose activity has a noteworthy effect on steering to network to our desired expression pattern. Thus, we extract the most effective external stimuli which have much effect on reducing the target functional value and our framework returns only these external stimuli that are really important. We increase α and perform the calculations where, for α=0.8, we only have $u_{3}$, $u_{6}$ and $u_{7}$ as active external stimuli. The results can be seen in Figure 12. This means that these three external stimuli, which are $u_{3}$ inhibits ERK1/2 dim 3P, $u_{6}$ activates angiotensin II and $u_{7}$ activates isoproterenol, are the important ones that we further investigate.

If we perform the same experiment just with the the active stimuli from Figure 12 for α=0 where we only use our combinatorial method (see Algorithm 1 in our Supplementary Materials), we then obtain $J_{0}=7.118559$ for a full activity of $u_{3}$ and $u_{7}$ (activity level of G${}_{s}$-coupled $\beta_{1}$ receptor about 98% of its maximum activation level) and $J_{0}=7.107087$ for full activity of $u_{3}$ and $u_{6}$ (activity level of G${}_{q}$-coupled ${\mathrm{AT}}_{1}$ receptor about 98% of its maximum activation level) which is almost the same target functional value as in the case with many external stimuli more shown in Figure 11. This demonstrates that this combination of external stimuli $u_{3}$ and $u_{7}$ or $u_{3}$ and $u_{6}$ are the essential ones to obtain a beneficial effect on the network, which means to have a low target functional value. While the other external stimuli also have beneficial effects, their contributions are minor compared to the effects of $u_{3}$ and $u_{7}$ or $u_{3}$ and $u_{6}$. We can say that compared with the value of the target functional of the unperturbed system ($J_{0}=160.1896)$ the strategies $u_{3}$ and $u_{7}$ or $u_{3}$ and $u_{6}$ are equivalent with the strategy shown in Figure 11 where the big advantage is that only two stimuli have to be applied instead of seven.

We remark without the external stimulus $u_{3}$, we are just able to obtain a target functional value of $J_{0}\approx 89$, which stresses the importance of the inhibition of ERK1/2 dim 3P for achieving of a beneficial state for the network.

#### 2.2.4. A Threshold for ERK Signaling

**Figure 13:**
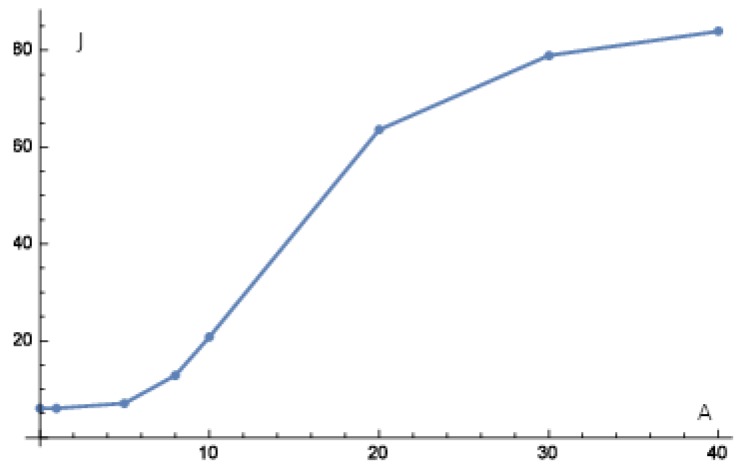
On the abscissa the activity level (A) of ERK1/2 dim 3P and on the ordinate the value (*J*) of $J_{0}$ is plotted. We see an abrupt increasing of the value of $J_{0}$ which can be interpreted as an abrupt deterioration of the corresponding treatment. Data points are from Table 2.

Next, we look at the sensitivity of the ERK signaling pathway. We investigate to what activity level ERK1/2 dim 3P has to be knocked down, i.e., has to be reduced, in order to be still as good as just inhibiting $G_{s}$-coupled $\beta_{1}$ receptor, that means to obtain a target functional value $J_{0}\approx 90$. We take the control strategy that $u_{3}$ inhibits ERK1/2 dim 3P and $u_{6}$ activates angiotensin. For this purpose, we use our combinatorial method (see Algorithm 1 in our Supplementary Materials), for different values of $\zeta_{22,3}$ which models the strength how much the activity level of ERK1/2 dim 3P (node 22) can be inhibited by the external stimulus $u_{3}$. The results are presented in Table 2, where for each experiment the external stimuli are fully active. We see that if the activity level of ERK1/2 dim 3P is at least at 10% of its maximum activity then, we still have a small target functional value $J_{0}\approx 20$ compared with $J_{0}\approx 90$ which is achieved by just applying our β-block strategy mentioned above. Furthermore, as the activity level of p90RSK and p70S6K are at 1 for all $\zeta_{22,3}$ in Table 2, we have that at about 5% of the maximum activity level of ERK 1/2 dim 3P the maleficent effects abruptly increase (activity levels of Elk1, MSK1 and c-Myc) which can be associated with an abrupt worsening of the treatment. For example, if one has a further restriction such as that the activity level of Elk1, MSK1 and c-Myc is supposed to be below 15%, then one can see from Table 2 that the activity level of ERK 1/2 dim 3P is supposed to stay below 5% of its maximum activity level. This can also be seen in Figure 13 where $J_{0}$ abruptly increases at 5% of ERK12 dim 3P maximum activity level. The interpretation is that the treatment abruptly worsens at 5% of ERK1/2 dim 3P maximum activity level. However, it does not mean that the treatment is already worse since higher values of $J_{0}$ can be tolerable in vivo such that only the beneficial effects are present while the maleficent effects are still not noticeable.

Additionally we still have a high activity level of p90RSK and p70S6K in contrast the strategy where one inhibits just the G${}_{s}$-coupled $\beta_{1}$ receptor. Furthermore, we see in Table 2 that the more we are able to inhibit ERK1/2 dim 3P the better it is for the treatment, i.e., the lower are the activity levels of Elk1, MSK1 and c-Myc.

**Figure 14:**
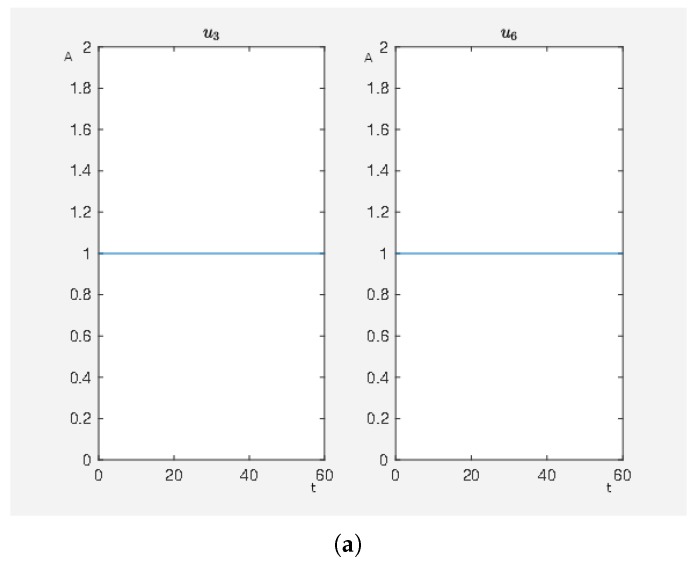

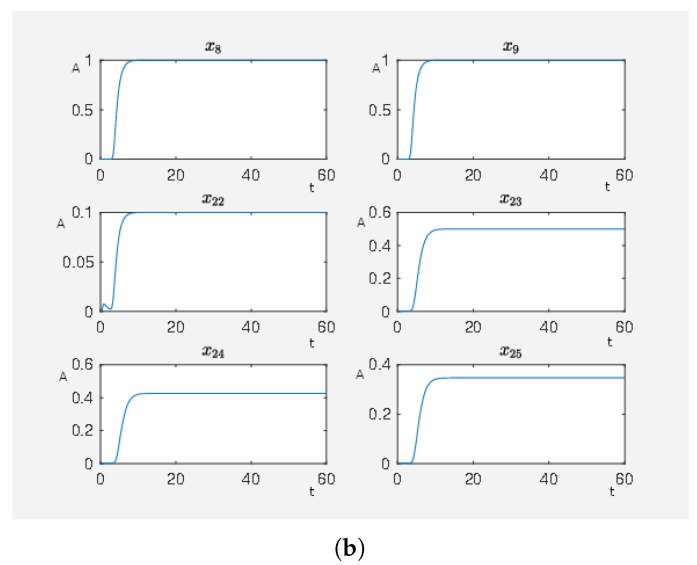
Activity levels for a concentration of three times phosphorylated ERK1/2 at 10% of its maximum concentration. (**a**) Time curve of external stimuli where $u_{3}$ inhibits ERK1/2 dim 3P, $u_{6}$ activates angiotensin II. On the abscissa, we have the time (t) and on the ordinate the activity level (A). (**b**) Time curve of $x_{8}$ the activity level of p90RSK, $x_{9}$ the activity level of p70S6K, $x_{22}$ activity level of ERK1/2 dim 3P, $x_{23}$ the activity level of Elk1, $x_{24}$ the activity level of MSK1 and $x_{25}$ the activity level of c-Myc. On the abscissa, we have the time (t) and on the ordinate the activity level (A).

In Figure 14, we can see the corresponding time curve for the activity level of ERK1/2 dim 3P at 10% of its maximum level. In this case, the activity level of p90RSK and p70S6K is 1 and of Elk1, MSK1 and c-Myc is between 0.3 and 0.5. We conclude that if the activity levels of p90RSK and p70S6K are low in spite of a constitutively activated G${}_{s}$-coupled $\beta_{1}$ receptor, one can recommend to activate the G${}_{s}$-coupled $\beta_{1}$ receptor even more by angiotensin II until the activity levels of p90RSK and p70S6K are high if one can manage it at the same time to inhibit the third phosphorylation of ERK1/2.

## 3. Discussion

The ERK signaling pathway is not just a pathway. To understand and treat it in diseases we have to look at the whole ERK network. ERK dimerization inhibitors or the manipulation of RKIP or GRK2 are all different ways that affect the ERK signaling network, especially with the purpose to help patients who suffer from heart insufficiency as soon as possible with better drugs. However, the effects of the pharmacological manipulation are complex and sometimes even counter intuitive on the first glance and not easy to understand. We know that the road to drugs for clinical use is still long, however, our current experiments illustrate that the targets discussed around ERK are of high therapeutical potential and merit more attention.

For this purpose, we now have developed a mathematical approach in order to calculate these effects and to make them visible. This approach is even applicable to other networks and in particular other cell types but we stress of course the central importance of ERK for the signal processing in the discussed example, the cardiomyocyte. The mathematical framework is explained in detail in the Supplementary Materials and is available for download in addition to the Matlab executables.

Of note, this model does not include all available information of the Ras/Raf/MEK/ERK1/2 signaling pathway but it can be easily modified by each researcher for the application in a certain cell type or certain signaling components. This could be the inclusion of phosphatases, inhibitor treatment or further signaling components as for example the study of a ERK1 mutant (ERK1${}^{R84S}$) that autophosphorylates at Thr207, which corresponds to Thr208 in mouse ERK2, but also seems to induce rather adaptive but maladaptive hypertrophy [26].

The mathematical framework is introduced and algorithms for the calculations are given. The usage is demonstrated based on a network for a cardiomyocyte where different treatment strategies are quantified with respect to beneficial and maleficent effects and evaluated based on the provided framework. Furthermore, it is shown how to model constitutively activated signal pathways causing a pathogenic state and different intervention points are systematically investigated to obtain an optimal drug combination.

Our model can be used for inhibition of the ERK cascade in cardiomyocyte hypertrophy (see Figure 13) and other pathologies (see Figure 9) for effects of c-Myc. The model can moreover be used to identify ERK as a promising drug target (see Figure 12) as well as to calculate to what degree ERK has to be inhibited to produce a comparable good treatment strategy as a non-hypertrophic stimulus.

It is shown how mathematical optimization can be used to analyze regulatory networks to determine drug combinations that have maximal beneficial effect while reducing side effects at the same time.

The method is a general framework. Although the example is performed with a cardiomyocyte model, the framework and the workflow is analogous for different models describing a biological experiment and may thus be interesting for the application in other diseases where the Ras/Raf/MEK/ERK1/2 cascade is involved in such as cancer, Parkinson, Rasophathies [11,27,28]. This also includes that the framework can be used for different cell types and is not restricted to the model of a cardiomyocyte. Any model set up of ordinary differential equations and thus any cell type can be considered, such as T-cells [29,30]. Further we can model lung cells [19], colon or liver cells [31]. In a similar way, any cell type can be considered provided sufficient information on network topology and interaction parameters is available such as in the ERK cascade. These models are fitted to experimental data, analogous to the model used in the presented work and thus the information contained in the fitted model parameters can be revealed by our framework as shown.

We note two further applications of the proposed framework. The first application is that we can test if a model is reasonable by applying different external stimuli and check if the network behaves as supposed according to experimental data including suggested alternative network topologies according to latest experimental data. The second application is as follows. An interaction graph can be set up where the governing ODE model can be fitted to real data created by the omics technology. All the possibilities of intervention by drugs can be modeled by external stimuli which can affect even more than just one node if one drug has multi target effects. Then, by our optimization framework, one can calculate the most effective drug combination that brings the activation level of the nodes of interest as close to the desired activity level as possible (see the Supplementary Materials, starting on the beginning of Page 4 for further details about this process).

We would like to stress that the limitation of the framework is in the model used. We cannot obtain more information from the model than is encoded in it. However, a model fitted to experimental data can contain a lot of hidden information and our general framework can help a lot to analyze the information with respect to answer a certain question. In our case that is find effective drug combination and pharmacological intervention points. In the Results Section, besides special results for ERK signaling, we also aim at showing how to use our framework in several generic situations, which is also a result in our opinion. To summarize the main points, we have a conclusion in the following.

## 4. Materials and Methods

**Figure 15:**
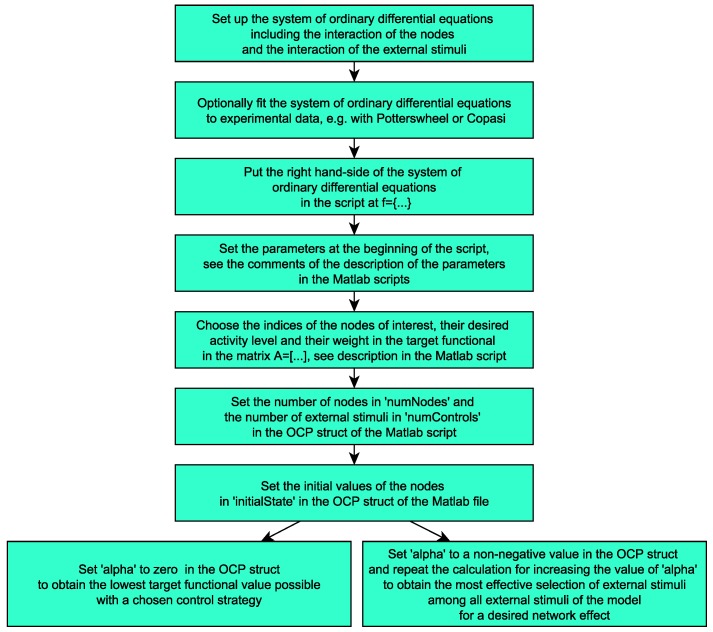
The workflow to use the provided script and the presented framework for the calculation of pharmacological intervention points and the comparison of treatment strategies. See the comments of the Matlab scripts for more details for its use and the setting of the parameters.

The calculations in this work were performed with the Matlab scripts provided for download (archive “Codes_optimal_pharmacological_intervention.zip”). Moreover, the framework can also be generalized for other signaling cascades, as explained in detail in the Supplementary Materials. A Matlab version with the SymbolicMath toolbox is required and the ParallelComputing toolbox is recommended. The scripts are commented and a readme file is given in which the function of the single scripts is described. The main file main_comp_therapies.m is set such that the calculations for the results depicted in Figure 4 are performed if executed with Matlab. The main file main_effective_treatment.m is set such that the calculations for the results depicted in Figure 12 are performed if executed with Matlab. Both files can easily be adapted to obtain the other results by setting the weight α in the OCP struct and by inserting external stimuli in the equations for the corresponding node. If in addition the right hand-side *f* in the script is replaced by different model equations, the scripts can be used for analogous calculations with other models to find and investigate optimal treatment strategies. We summarize the workflow to use the provided Matlab scripts and the provided framework for the calculation of pharmacological intervention points and the comparison of treatment strategies in the following flow chart (Figure 15).

As mentioned, we provide the Matlab scripts. The framework and the scripts are elaborated such that the user does not have to be able to program Matlab. The only thing that has to be done to use the provided script for any model within the framework is to change the formulas for the model. This is done where f = {...} is written in the Matlab scripts. Then, the parameters in the beginning of the Matlab script of the “OCP” struct can be changed, which also needs no programming skills in Matlab.

## 5. Conclusions

Using the well-studied and central ERK cascade of the cardiomyocyte, we showed that we can study its sensitivity, network effects, crosstalk threshold effects and treatment strategies.

The sensitivity of the ERK cascade was studied under external stimuli. For this purpose, the effectiveness of a dimerization inhibitor was reduced (see the experiment associated with Figure 13).

Network effects for ERK signaling were considered in the experiments associated with Figure 3, Figure 4, Figure 5 and Figure 6, which show how the influence of certain nodes affects the activity of the ERK signaling.

Threshold effects in the ERK signaling cascade were investigated. In the experiment associated with Figure 9, it was shown that a certain activity level of the dimerization of ERK, just a small reduction of the activity level, results in a reduction of the nuclear agents Elk1, MSK1 and c-Myc chosen for demonstration. Our simplified models explore the ERK cascade. More complex effects (considering more nuclear agents, more phosphorylation substrates, indirect interactions, modifiers, and cross-talk) might be considered for extending the scripts provided in the paper to more refined models.

As a general approach for the search for pharmacological intervention points, the following points are contained in the presented paper.

In Section 2.1, the proposed framework is used to compare different treatment strategies.

In the experiment associated with Figure 12, it was shown how to use the proposed framework to find automatically and systematically the most effective treatment strategy out of a given number of external stimuli, exemplified on a constitutively activated $G_{s}$-coupled $\beta_{1}$ receptor.

## Figures and Tables

**Table 1:** Parameters for the network shown in Figure 2 according to the model (2.1) in the Supplementary Materials.

*k*	Name of the Node	$\omega_{k}$
1	non-hypertrophic stimulus	
2	carbachol	$\frac{11x_{1}}{1+10x_{1}}$
3	$G_{i}$-coupled $M_{2}$ receptor	$\frac{11x_{2}}{1+10x_{2}}$
4	Ras (GTP bound)	$\frac{\frac{13}{12}\left(10x_{3}+x_{12}+x_{19}\right)}{1+10x_{3}+x_{12}+x_{19}}$
5	Raf1	$\frac{11x_{4}}{1+10x_{4}}\left(1-\frac{1.01x_{14}}{1+0.01x_{14}}\right)$
6	MEK1/2	$\frac{2x_{5}}{1+x_{5}}$
7	ERK1/2 dim 2P	$\frac{1.001x_{6}}{1+0.001x_{6}}$
8	p90RSK	$\frac{1.01x_{7}}{1+0.01x_{7}}$
9	p70S6K	$\frac{1.01x_{7}}{1+0.01x_{7}}$
10	hypertrophic stimulus	
11	angiotensin II	$\frac{2x_{10}}{1+x_{10}}$
12	$G_{q}$-coupled ${\mathrm{AT}}_{1}$ receptor	$\frac{6x_{11}}{1+5x_{11}}$
13	PKC	$\frac{\frac{31}{30}\left(10x_{3}+10x_{12}+10x_{20}\right)}{1+10x_{3}+10x_{12}+10x_{20}}$
14	RKIP	$\frac{2x_{26}}{1+x_{26}}\left(1-\frac{11x_{13}}{1+10x_{13}}\right)$
15	RKIP dim	$\frac{11x_{13}}{1+10x_{13}}$
16	GRK2	$\frac{2x_{26}}{1+x_{26}}\left(1-\frac{31x_{15}}{1+30x_{15}}\right)$
17	AND	$\frac{2x_{26}}{1+x_{26}}\left(1-\frac{101x_{7}}{1+100x_{7}}\right)$
18	isoproterenol	$\frac{11x_{10}}{1+10x_{10}}$
19	$G_{s}$-coupled $\beta_{1}$ receptor	$\frac{11x_{18}}{1+10x_{18}}\left(1-\frac{1.1x_{16}}{1+0.1x_{16}}\right)$
20	Epac	$\frac{11x_{19}}{1+10x_{19}}$
21	$G_{\beta \gamma }$	$\frac{\frac{21}{20}\left(10x_{12}+10x_{19}\right)}{1+10x_{12}+10x_{19}}$
22	ERK1/2 dim 3P	$\frac{101x_{21}}{1+100x_{21}}\left(1-\frac{2x_{17}}{1+x_{17}}\right)$
23	Elk1	$\frac{9x_{22}}{1+8x_{22}}$
24	MSK1	$\frac{8x_{22}}{1+7x_{22}}$
25	c-Myc	$\frac{7x_{22}}{1+6x_{22}}$
26	SYSTEM STATE	

**Table 2:** Activity levels of Elk1, MSK1 and c-Myc for increasing inhibition of the third ERK1/2 phosphorylation.

$\zeta_{22,3}$	$J_{0}$	Activity Level in %			
		ERK 1/2 dim 3P	Elk1	MSK1	c-Myc
1	6.08	0	0	0	0
0.99	6.08	1	1	1	1
0.95	7.12	5	14	11	8
0.92	12.89	8	35	29	22
0.9	20.81	10	50	43	35
0.8	63.57	20	88	85	80
0.7	78.90	30	96	95	93
0.6	83.98	40	98	97	97

## Supplementary Materials

The supplementary manuscript with the mathematical and the algorithmic details are available online at https://www.mdpi.com/1422-0067/20/9/2179/s1. The Matlab implementations are available at https://www.mdpi.com/1422-0067/20/9/2179/s2.
