# Supplementary material for: How to Steer and Control ERK and the ERK Signaling Cascade Exemplified by Looking at Cardiac Insufficiency
Source: Int J Mol Sci. 2019 May 2;20(9):2179. doi: 10.3390/ijms20092179 (PMC6539830; doi:10.3390/ijms20092179)
Supplement: Supplementary file 1 [file ijms-20-02179-s001.pdf]

Supplementary material to How to steer and control ERK and the ERK signalling cascade exemplified by looking at cardiac insufficiency

In the supplementary material we explain the mathematical framework in detail that is used for the calculations. In addition we give details about the used algorithms.

1 Introduction

The mathematical modeling of molecular biological systems is often done by regulatory networks. For this purpose equations as in [11] can be used for instance. In a next step, the regulatory network is analyzed with respect to its steady states, see [5, 8] in order to find stable expression patterns in the network. This concept of regulatory networks is extended by external stimuli to model the observation that external stimuli can change the expression level of certain genes and thus transfer the system from one stable expression pattern to another one, see [2]. That means, although the external stimuli are not active any more, the network remains in its steady state which it has been steered to by the external stimuli. However, there are systems, which change their expression pattern just as long as the external stimuli are active. When the external stimuli are not present any more, then the system relaxes back to the "ground state" which it left because of the perturbation by the external stimuli. Examples for such a framework is when a pathogen senses its host by certain agents [3, 6, 7, 16] or other pathogens [20, 12], called quorum sensing. These agents differ in different environments and thus serve as signals or in our framework external stimuli which change the gene expression of a bacterium or pathogen for instance. Further external stimuli are physical ones like temperature [19], mechanical stress [4, 18, 10] or gravity [17]. Also starvation can lead to cell cycle arrest [15] and changes the expression pattern [14, 1] and thus acts as an external stimulus where the expression pattern changes as soon as the stimulus decays. Also communication between cells can be modeled. For example the secretion of interferon by T-cells can change the expression pattern of other immune cells as long as interferon is present [9]. For this purpose, one can make usage of the advantage of our presented framework and model each cell with its own network where the activity of certain nodes of the first cell can be associated with the secretion of agents that can serve as external stimuli for the second cell.

Another external stimulus can be a pharmacological affection of a network in order to change the expression pattern of a cell from a pathological state to a favorable state. Our framework fits well to the case, when after the treatment and the decay of the pharmacological active agents, the network relaxes back to the pathological state. This is different to [2], where the network stays in its desired state although the pharmacological active agents decay because the treatment steered the network to a new stable state.

An optimal expression level is that we change just the pathologically expressed genes while leaving the normally expressed genes unperturbed to reduce side effects. In order to achieve such an expression pattern we set up a framework to calculate optimal drug targets to affect the network in our favor. For this purpose, we define a target functional which has a small value if the nodes of interest of a regulatory network are close to their desired expression level, that means high beneficial effects and low side effects. Within this framework, we can now evaluate different treatment strategies, that is which nodes of the network are to be affected by external stimuli, and sort them with respect to their beneficial and maleficent effects. In order to identify rewarding and effective drug targets, we use a mathematical optimization framework where we minimize the mentioned target functional subject to the constraint given by a system of ordinary differential equations which is used to model the interaction of the

network's nodes. Furthermore, by this mathematical framework, the proposed method is objective as the external stimuli are determined such that a fixed target functional is minimized. Additionally, the optimization framework provides that we just have to care about which nodes the stimuli are supposed to act on while the rest, like time curve of each external stimulus, is determined by the software such that the target functional is minimized. The consequence is that the result is the lowest target functional value which we can achieve with the chosen treatment strategy which ensures the comparability of different strategies.

This framework is a rational method to calculate candidates of drugs or combinations of them with good prospect to have the desired effects on the network with low side effects at the same time. Furthermore, the proposed method is a systematic way to find promising drug targets for a treatment and the tightly focused development of new medicaments which cause a desired expression level of genes. The proposed framework can be used as a preliminary study in silico in order to focus on promising experiments in vitro or in vivo. This especially means that new drugs can be developed where the only experiments which have to be made are the ones to prove the desired effect of the new drug and the experiments used to find candidates which might have the desired effect can be fully made in silico. This is particularly useful if one studies huge networks (more than 150 nodes) that are made of the information of an interactom, for instance, in combination with all the drugs that are available to interact on this network where one drug can even have several nodes to interact. By this framework we have a powerful tool to exploit all the information coded within this interaction graph, which can be fitted to real data by omics technology, to calculate the most effective drug combination. That means maximizing beneficial effects while reducing side effects at the same time.

In the following section, we give the mathematical changes needed to adapt the detailed discussion of [2, Supplement Section 1] to the proposed framework here. Furthermore, we discuss a combinatorial algorithm to determine optimal drug targets. In Section 3, we show that this framework presented in this work can be used for the analysis of external stimuli to switch between different steady states. We show that the results are in accordance with previous work.

2 The mathematical framework

In this section, we introduce a mathematical model for the optimal control of regulatory networks where only the activity level of certain nodes is of interest and not necessarily the expression pattern of the whole network. The activity level of a node associated with a gene corresponds to the transcription rate of RNA or to translation rate of the corresponding protein. If the node is a protein, then the activity stands for the production rate for an associated agent related to available reactant. In general the activity level of a node is the biological activity of the associated biological agent within a known natural range where the lowest activity level is associated with inactivity and the highest activity level with the highest biological activity that is biological reasonable and observable. An application of this framework is optimal drug targeting which is discussed in this work. We assume that the different agents of interests of a real (biological) network are modeled by a regulatory network. Each agent is associated with a node $k \in \{1, ..., n\}$ where $n \in \mathbb{N}$ is the number of nodes. The activity level $x_k : \mathbb{R}_0^+ \to [0,1]$ of the each node k with the initial value $x_k(0) = x_k^0 \in [0,1]$ is calculated by

$$\frac{dx_k}{dt} = \frac{-\mathrm{e}^{\frac{1}{2}h} + \mathrm{e}^{-h\left(\omega_k - \frac{1}{2}\right)}}{\left(1 - \mathrm{e}^{\frac{1}{2}h}\right)\left(1 + \mathrm{e}^{-h\left(\omega_k - \frac{1}{2}\right)}\right)} \prod_{j=1}^{m} \left(1 - \zeta_{kj} u_j\right) - \gamma_k x_k + \sum_{j=1}^{m} \sigma_{kj} u_j \left(1 - x_k\right) \tag{2.1}$$

until a final time $T > 0$ with

$$\omega_k = \begin{cases} EG & \text{if } x_k \text{ has activators and inhibitors} \\ E & \text{if } x_k \text{ has only activators} \\ G & \text{if } x_k \text{ has only inhibitors} \end{cases}$$

where

$$E = \left(\frac{1 + \sum_{j\in A_k} \alpha_j^k}{\sum_{j\in A_k} \alpha_j^k}\right)\left(\frac{\sum_{j\in A_k} \alpha_j^k x_j}{1 + \sum_{j\in A_k} \alpha_j^k x_j}\right), \quad G = \left(1 - \left(\frac{1 + \sum_{j\in I_k} \beta_j^k}{\sum_{j\in I_k} \beta_j^k}\right)\left(\frac{\sum_{j\in I_k} \beta_j^k x_j}{1 + \sum_{j\in I_k} \beta_j^k x_j}\right)\right)$$

the activators of node k are elements of the subset $\{x_j|\ j \in A_k \subseteq \{1,...,n\}\} \subseteq \{x_k|\ k \in \{1,...,n\}\}$ where A_k contains all the indices of $\{1,...,n\}$ of the nodes which activate node k and the corresponding $\alpha_j^k > 0$ weights the contribution of the activation level x_j of node j to the total activation of node k. Analogously, the inhibitors of node k are elements of the subset $\{x_j|\ j \in I_k \subseteq \{1,...,n\}\} \subseteq \{x_k|\ k \in \{1,...,n\}\}$ where I_k contains all the indices of $\{1,...,n\}$ of the nodes which inhibit node k and the corresponding $\beta_j^k > 0$ weights contribution of activation level x_j of node j to the total inhibition of node k. Furthermore, the gain $h > 0$ can be seen as a measure of cooperativity that means how abruptly the activation level of node k changes if the input values reach a certain threshold value. The decay $\gamma_k > 0$ for all $k \in \{1,...,n\}$ models how fast the activity level of node k decays if there is no input for it.

This model is based on [11] and is extended by the framework of external stimuli and further discussed in [2]. The set of external stimuli is given by $S := \{u_j|\ j \in \{1,...,m\}\}$, $m \in \mathbb{N}$ where $u_j : \mathbb{R}_0^+ \to [0,1]$ are functions. These external stimuli effect the activity level x_k of a node k either by activation or inhibition. The activation is is given in the last term of (2.1). The parameter σ_{kj} weights the coupling of the node x_k and the external stimulus j. If $\sigma_{kj} = 0$, then there is no effect of external stimulus u_j on x_k. By the parameter σ_{kj} we can adjust the model such that the activation level x_k calculated in the model fits to the observed activation level of the corresponding agent in an experiment when the corresponding external stimulus is applied to activate this agent with a certain intensity u_j modeled by the values between 0 and 1 where 0 means no application of external stimuli j and 1 with the highest intensity which is possible or reasonable.

The inhibition is modeled by the term $\prod_{j=1}^{m}(1-\zeta_{kj}u_j)$ where $\zeta_{kj} \in [0,1]$ models the influence of external stimulus j on node k. If $\zeta_{kj} = 0$, then external stimulus j has no effect on node k. Depending on u_j and ζ_{kj}, $k \in \{1,...,n\}$, $j \in \{1,...,m\}$, the first term of (2.1), the activation function, is multiplied by a factor between 0 and 1 coming from $\prod_{j=1}^{m}(1-\zeta_{kj}u_j)$. This reduces the value of the activation function and results that the decay reduces the activation level of the corresponding node. If $\sigma_{kj} = 1$ and if there is a full activation of the corresponding external stimulus j, i.e. $u_j = 1$, then the activation function is zero. By the parameter ζ_{kj} we can adjust the model such that the activation level x_k calculated in the model fits to the observed activation level of the corresponding agent in an experiment when the corresponding external stimulus is applied to inhibit this agent with a certain intensity u_j modeled by the values between 0 and 1 where 0 means no application of external stimuli j and 1 with the highest intensity which is possible or reasonable.

Remark 1. We remark that this method proposed in our work is in principle not restricted to that specific model 2.1. The proposed method can be equipped with any well-posed system of ordinary differential equations with which one would like to model a real system.

Now, once we have modeled the dynamics of the network, we have desired activation levels for certain nodes. These desired activation levels $x_k^d : \mathbb{R}_0^+ \to [0,1]$ are functions which represent the desired activation level of node k. These functions can be constant or vary their value over time. The set of desired activation levels is defined by $D := \left\{x_k^d|\ k \in N_I\right\}$, $N_I \subseteq \{1,...,n\}$. Notice that the proposed method works even if we are just interested in the activation level of a subset of nodes of the whole network which means that the cardinality of D is less then n.

The task is now to determine external stimuli from a given set S, and if necessary their temporal curve, such that each node of interest from the set N_I takes its desired value as well as possible. We define $x := \begin{pmatrix} x_1 \\ \vdots \\ x_n \end{pmatrix}$ and $u := \begin{pmatrix} u_1 \\ \vdots \\ u_m \end{pmatrix}$ and the following target functional

$$\tilde{J}(x,u) := \frac{1}{2}\sum_{k=1}^{n} g_k \int_0^T \left(x_k(t) - x_k^d(t)\right)^2 dt,$$

where $g_k \in \mathbb{R}_0^+$ is the weight of the corresponding tracking term $\int_0^T \left(x_k(t) - x_k^d(t)\right)^2 dt$ with $g_k > 0$ if $k \in N_I$ and $g_k = 0$ else. The weights relatively weight how important it is that the corresponding node achieves its desired value compared to the other nodes of interest. The greater the value g_k for

a certain node k is compared to the other nodes of interest, the more important it becomes that the corresponding node k attains its desired state x_k^d compared with the other nodes where $g_k > 0$.

An application can be as follows. An interaction graph can be set up where the governing ODE model can be fitted to real data created by the omics technology. All the possibilities of intervention by drugs can be modeled by external stimuli which can affect even more than just one node if one drug has multi target effects. Then by our optimization framework one can calculate the most effective drug combination that brings the activation level of the nodes of interest as close to the desired activity level as possible. In general it will be not possible the meet the desired state exactly. If the user wishes to have some certain nodes closer to a desired value as in the calculated solution because they are still to far away from a desired value such that it is physiologically still not reasonable, they can increase the corresponding g_k and perform the calculations again. Then these nodes might get closer to the desired state, however maybe at the cost that others might get away a little bit more form its desired state but still close enough such that the new expression pattern caused by the external stimuli makes physiologically sense. If the expression pattern is still not as close to the desired one then one has to include more external stimuli and thus can identify new effective drug targets that have the desired effect. This demonstrates how the optimization framework can be used to extract promising drug combinations out of a huge graph containing all the available information that steer the experiment close to a physiological desired expression pattern.

Now, we can measure by the value of $\tilde{J}(x,u)$ how well the desired activity levels are taken by their corresponding nodes of interest subject to the constraint that (x,u) fulfills (2.1). That means the smaller $\tilde{J}$ is the better the desired activation levels are taken where in the best case $\tilde{J} = 0$ which means that $x_k = x_k^d$ for all $k \in N_I$. In order to include costs of the external stimuli into the target functional, we add the term $\alpha \sum_{j=1}^m \int_0^T u_j(t)\,dt$, $\alpha \geq 0$ which extracts the most effective external stimuli. This works as follows. As for large values of α it is more likely that the cost of an active external stimuli is greater than its effect on steering the nodes of interest to the desired activation level and thus is set to zero. Then, we have the following extended cost functional

$$J_\alpha(x,u) := \frac{1}{2}\sum_{k=1}^n g_k \int_0^T \left(x_k(t) - x_k^d(t)\right)^2 dt + \alpha \sum_{j=1}^m \int_0^T u_j(t)\,dt.$$

Notice that $J_0(x,u) = \tilde{J}(x,u)$ Summarizing, we now have the following problem

$$\begin{aligned} &\min_{y,u} J_\alpha(x,u) \\ &\text{subject to } \frac{d}{dt}x_k = f_k(x,u) \text{ for all } k \in \{1,...,n\} \end{aligned} \tag{2.2}$$

where $f_k(x,u) := \frac{-e^{\frac{1}{2}h} + e^{-h\left(\omega_k - \frac{1}{2}\right)}}{\left(1-e^{\frac{1}{2}h}\right)\left(1+e^{-h\left(\omega_k - \frac{1}{2}\right)}\right)} \prod_{j=1}^m (1 - \zeta_{kj}u_j) - \gamma_k x_k + \sum_{j=1}^m \sigma_{kj}u_j(1-x_k)$ is given by the right hand-side of (2.1).

An approach for solving (2.2) is to systematically try different external stimuli with constant value over the interval $[0,T]$ such that there might at least be one combination that generates a smaller target functional value than the constant zero which means that no external stimuli is active. This idea is implemented in Algorithm 1 as follows. We choose the maximum number of active stimuli $\mathrm{maxNum} \in \{1,...,m\}$ and the number $\mathrm{numInt} \in \mathbb{N}$ in which the image of the external stimuli $[0,1]$ is divided into. Then we choose from the power set $\mathcal{P}$ of the set $\{1,...m\}$ that elements p with cardinality $|p| \leq \mathrm{maxNum}$. This defines the set P. Next, we choose $p \in P$ and $val \in \left\{i\frac{1}{\mathrm{numInt}} \middle| \, i = 1,...,\mathrm{numInt}\right\}$ and calculate the target functional value $J_\alpha(x,u)$ for the corresponding external stimuli u where $u_j = val$ if $j \in p$ and $u_j = 0$ else and(x,u) fulfills (2.1). This is done for all $p \in P$ and all $val \in \left\{i\frac{1}{\mathrm{numInt}} \middle| \, i = 1,...,\mathrm{numInt}\right\}$.

Algorithm 1 Combinatorial method

1. Choose numMax $\in \{1,...,m\}$, numInt $\in \mathbb{N}$
2. Choose all the elements $p \in P \subseteq \mathcal{P}$ of the power set $\mathcal{P}$ of $\{1,...,m\}$ such that the cardinality $|p| \leq$ maxNum
3. Calculate $J_\alpha(x,u)$ for all $p \in P$ and all $val \in \left\{i\frac{1}{\text{numInt}} \mid i = 1,...,\text{numInt}\right\}$ with $u_j = val$ if $j \in p$ and $u_j = 0$ else where (x,u) fulfills (2.1)
4. Return (x,u) with the smallest target functional value $J_\alpha(x,u)$

Notice that it is worth to parallelize an implementation of Algorithm 1 as the calculation for each combination (x,u) in step 3 is independent of the others. Furthermore, we remark the calculation time of Algorithm 1 increases exponentially with respect to maxNum.

The output of 1 can be used as an initial guess of a local optimization methods like the sequential quadratic Hamiltonian method, see [2]. Based on the initial guess from Algorithm 1, a local optimization method finds an optimal solution for (2.2) in almost linear calculation time with respect to the number of external stimuli. As the external stimuli are functions over time, the solution of a local optimization time is not necessary a constant function but its value can vary with time. The different time curves of the external stimuli corresponding to a minimum of (2.2) may contain further information about the relation of each external stimulus to each other. Additionally an optimal solution from a local optimization method can differ with respect to the number of active external stimuli compared with the solution from Algorithm 1. Notice that the calculation from local optimization algorithms might be more efficient due to the time scaling depending on the number of external stimuli.

In order to use the sequential quadratic Hamiltonian method [2, Algorithm 2], we just have to modify some definitions made in [2, Supplement].

The Hamiltonian $H : \mathbb{R} \times \mathbb{R}^n \times \mathbb{R}^m \times \mathbb{R}^n$ now is given by

$$H(t,x,u,p) := \frac{1}{2}\sum_{k=1}^{n} g_k \left(x_k - x_k^d\right)^2 + \alpha \sum_{j=1}^{m} u_j + \sum_{k=1}^{n} p_k f_k(x,u).$$

The adjoint variables $\bar{p}_k : \mathbb{R}_0^+ \to \mathbb{R}$ are given by the following adjoint equations

$$\frac{d\bar{p}_k}{dt} = -g_k\left(\bar{x}_k - x_k^d\right) - \sum_{i=1}^{n} \bar{p}_i \frac{\partial}{\partial x_k} f_i(\bar{x},\bar{u})$$

if $k \in N_I$ and by the following equation

$$\frac{d\bar{p}_k}{dt} = -\sum_{i=1}^{n} \bar{p}_i \frac{\partial}{\partial x_k} f_i(\bar{x},\bar{u})$$

if $k \in \{1,...,n\} \setminus N_I$ with $\bar{p}(T) = 0$ for all $k \in \{1,...,\}$ where $\frac{\partial}{\partial x_k} f_i(\bar{x},\bar{u}) := \frac{\partial}{\partial x_k} f_i(x,u)\,|_{(x,u)=(\bar{x},\bar{u})}$ that means the partial derivative of the i-th component of f with respect to the k-th component of x evaluated at $(\bar{x},\bar{u})$ and $(\bar{x},\bar{u})$ is an optimal solution to (2.2).

Alternatively, as in [2, Supplement], we can discretize (2.2) before we optimize with a local optimization method like the gradient method, see [2, Algorithmus 1] and obtain for a certain time step size $dt > 0$, $t = ldt$, $l \in \{0,...,N\}$ and $T = Ndt$ the following optimal control problem

$$\min_{x,u} J(x,u) := \frac{1}{2}\sum_{k=1}^{n} g_k \sum_{l=1}^{N} \left(x_k^l - x_k^d(ldt)\right)^2 dt + \alpha \sum_{j=1}^{m}\sum_{l=0}^{N-1} u_j^l dt$$
$$\text{subject to } F(x,u) = 0,\ x^0 = x_0$$

where $F(x,u) := \begin{pmatrix} F_1(x,u) \\ \vdots \\ F_n(x,u) \end{pmatrix} \in \mathbb{R}^{nN}$,

$$F_k(x,u) := -\begin{pmatrix} x_k^1 \\ \vdots \\ x_k^N \end{pmatrix} + \begin{pmatrix} x_k^0 \\ \vdots \\ x_k^{N-1} \end{pmatrix} + \begin{pmatrix} f_k\left(x^0,u^0\right) \\ \vdots \\ f_k\left(x^{N-1},u^{N-1}\right) \end{pmatrix} dt \in \mathbb{R}^N$$

for all $k \in \{1,...,n\}$, x_k is not a function any more but a vector $x_k := \begin{pmatrix} x_k^1 \\ \vdots \\ x_k^N \end{pmatrix} \in \mathbb{R}^N$ for all $k \in \{1,...,n\}$ and $x := \begin{pmatrix} x_1 \\ \vdots \\ x_n \end{pmatrix} \in \mathbb{R}^{nN}$. Analogously, we have the definition for the vector $u_j := \begin{pmatrix} u_j^0 \\ \vdots \\ u_j^{N-1} \end{pmatrix} \in \mathbb{R}^N$ and $u := \begin{pmatrix} u_1 \\ \vdots \\ u_m \end{pmatrix} \in \mathbb{R}^{mN}$ with $0 \leq u_j^l \leq 1$ for all $j \in \{1,...,m\}$ and $l \in \{0,...,N-1\}$. Then, we just have to modify the adjoint equation for $p := \begin{pmatrix} p_1 \\ \vdots \\ p_n \end{pmatrix} \in \mathbb{R}^{nN}$, $p_k := \begin{pmatrix} p_k^1 \\ \vdots \\ p_k^N \end{pmatrix} \in \mathbb{R}^N$ which is given by

$$0 = \begin{pmatrix} g_k\left(x_k^1 - x_k^d(1dt)\right) - p_k^1 + p_k^2 + \sum_{i=1}^n p_i^2 \frac{\partial}{\partial x_k^1} f_i\left(x^1,u^1\right) dt \\ g_k\left(x_k^2 - x_k^d(2dt)\right) - p_k^2 + p_k^3 + \sum_{i=1}^n p_i^3 \frac{\partial}{\partial x_k^2} f_i\left(x^2,u^2\right) dt \\ \vdots \\ g_k\left(x_k^{N-1} - x_k^d((N-1)dt)\right) - p_k^{N-1} + p_k^N + \sum_{i=1}^n p_i^N \frac{\partial}{\partial x_k^{N-1}} f_i\left(x^{N-1},u^{N-1}\right) dt \\ g_k\left(x_k^N - x_k^d(Ndt)\right) - p_k^N \end{pmatrix} \in \mathbb{R}^N$$

for all $k \in N_I$ and

$$0 = \begin{pmatrix} -p_k^1 + p_k^2 + \sum_{i=1}^n p_i^2 \frac{\partial}{\partial x_k^1} f_i\left(x^1,u^1\right) dt \\ -p_k^2 + p_k^3 + \sum_{i=1}^n p_i^3 \frac{\partial}{\partial x_k^2} f_i\left(x^2,u^2\right) dt \\ \vdots \\ -p_k^{N-1} + p_k^N + \sum_{i=1}^n p_i^N \frac{\partial}{\partial x_k^{N-1}} f_i\left(x^{N-1},u^{N-1}\right) dt \\ -p_k^N \end{pmatrix} \in \mathbb{R}^N$$

for all $k \in \{1,...,n\} \backslash N_I$.

3 Steady state switch in a platelet network and in a T-helper cell network

This short section is intended to show that the present framework can also be used to induce a switch between two different steady states of a network. We repeat the experiment from[2, Section 4] with the corresponding model to demonstrate that the presented framework also calculates the receptors that are associated with irreversible platelet aggregation if the receptors are activated. As known from [13] the irreversible platelet aggregation is associated with a high activity of integrin. We start with the steady state associated with the reversible platelet aggregation where the activity of integrin is

low and chose as the node of interest the node that is associated with integrin. We desire this node to have an activity level equal to 1. We use the result from Algorithm 1 for $\alpha = 0.01$, numMax $= 2$ and numInt $= 3$ as the initial guess for the external stimuli for [2, Algorithm 2] with the recommended parameters except $\kappa = 10^{-14}$. The constant non zero external stimuli from Algorithm 1 are set to zero at the interval $[0, T]$ from [2, Algorithm 2] and thus the network relaxes into the desired steady state. We obtain the results in accordance to [2, Figure 2 left one] which means that the system is in the desired steady state and the same external stimuli are active and the same are inactive.

We also repeat the experiment from [2, Section 5] with the corresponding model to induce a switch from the steady state that is associated with a Th-17 cell to the steady state that is associated with a regulatory Treg cell. The specifying lineage transcription factor for Treg is FOXP3. For this purpose, we choose only FOXP3 as our only node of interest and desire it to have the value 1. We use the result from Algorithm 1 for $\alpha = 0.1$, numMax $= 5$ and numInt $= 3$ for the initial guess for the external stimuli for [2, Algorithm 2] with the recommended parameters except $\kappa = 10^{-14}$ and obtain results in accordance to [2, Figure 4 left one]. This means that the network is in the same desired state at the end of the interval $[0, T]$ where the non zero external stimuli from Algorithm 1 are set to zero by [2, Algorithm 2] and still the same external stimuli are active and the same are inactive at the beginning of the interval $[0, T]$.

References

[1] Nick Bos, Unni Pulliainen, Liselotte Sundström, and Dalial Freitak. Starvation resistance and tissue-specific gene expression of stress-related genes in a naturally inbred ant population. *Royal Society open science*, 3(4):160062, 2016.

[2] Tim Breitenbach, Chunguang Liang, and Thomas Dandekar. Analyzing pharmacological intervention points: A method to calculate external stimuli to switch between steady states in regulatory networks. 2019.

[3] Stéphane Bronner, Henri Monteil, and Gilles Prévost. Regulation of virulence determinants in staphylococcus aureus: complexity and applications. *FEMS microbiology reviews*, 28(2):183–200, 2004.

[4] Matthias Chiquet. Regulation of extracellular matrix gene expression by mechanical stress. *Matrix biology*, 18(5):417–426, 1999.

[5] Alessandro Di Cara, Abhishek Garg, Giovanni De Micheli, Ioannis Xenarios, and Luis Mendoza. Dynamic simulation of regulatory networks using squad. *BMC bioinformatics*, 8(1):462, 2007.

[6] Primrose Freestone. Communication between bacteria and their hosts. *Scientifica*, 2013, 2013.

[7] Donald G. Guiney. Regulation of bacterial virulence gene expression by the host environment. *The Journal of clinical investigation*, 99(4):565–569, 1997.

[8] Stefan Karl and Thomas Dandekar. Jimena: efficient computing and system state identification for genetic regulatory networks. *BMC bioinformatics*, 14(1):306, 2013.

[9] Stefan H.E. Kaufmann and Inge E.A. Flesch. The role of T cell-macrophage interactions in tuberculosis. In *Springer seminars in immunopathology*, volume 10, pages 337–358. Springer, 1988.

[10] Coralia Luna, Guorong Li, Paloma B. Liton, David L. Epstein, and Pedro Gonzalez. Alterations in gene expression induced by cyclic mechanical stress in trabecular meshwork cells. *Molecular vision*, 15:534, 2009.

[11] Luis Mendoza and Ioannis Xenarios. A method for the generation of standardized qualitative dynamical systems of regulatory networks. *Theoretical Biology and Medical Modelling*, 3(1):13, 2006.

[12] Samuel I. Miller, Lucas R. Hoffman, and Sarah Sanowar. Did bacterial sensing of host environments evolve from sensing within microbial communities? *Cell host & microbe*, 1(2):85–87, 2007.

[13] Marcel Mischnik, Stepan Gambaryan, Hariharan Subramanian, Jörg Geiger, Claudia Schütz, Jens Timmer, and Thomas Dandekar. A comparative analysis of the bistability switch for platelet aggregation by logic ODE based dynamical modeling. *Molecular BioSystems*, 10(8):2082–2089, 2014.

[14] Kazuyuki Shimizu. Regulation systems of bacteria such as escherichia coli in response to nutrient limitation and environmental stresses. *Metabolites*, 4(1):1–35, 2013.

[15] Jae-Sik Shin, Seung-Woo Hong, Sae-Lo Oom Lee, Tae-Hee Kim, In-Chul Park, Sung-Kwan An, Won-Keun Lee, Jong-Seok Lim, Keun-Il Kim, Young Yang, et al. Serum starvation induces g1 arrest through suppression of skp2-cdk2 and cdk4 in sk-ov-3 cells. *International journal of oncology*, 32(2):435–439, 2008.

[16] Mark S. Thomas and Sivaramesh Wigneshweraraj. Regulation of virulence gene expression. *Virulence*, 5(8):832–834, 2014.

[17] Raja Vukanti, Eric Mintz, and Laura Leff. Changes in gene expression of E. coli under conditions of modeled reduced gravity. *Microgravity-Science and Technology*, 20(1):41, 2008.

[18] James H.-C. Wang, Bhavani P. Thampatty, Jeen-Shang Lin, and Hee-Jeong Im. Mechanoregulation of gene expression in fibroblasts. *Gene*, 391(1):1–15, 2007.

[19] Gregor G. Weber, Jens Kortmann, Franz Narberhaus, and Karl E Klose. Rna thermometer controls temperature-dependent virulence factor expression in vibrio cholerae. *Proceedings of the National Academy of Sciences*, 111(39):14241–14246, 2014.

[20] Klaus Winzer and Paul Williams. Quorum sensing and the regulation of virulence gene expression in pathogenic bacteria. *International Journal of Medical Microbiology*, 291(2):131–143, 2001.
